# Comparison of Vacuum MALDI and AP-MALDI Platforms for the Mass Spectrometry Imaging of Metabolites Involved in Salt Stress in *Medicago truncatula*

**DOI:** 10.3389/fpls.2018.01238

**Published:** 2018-08-28

**Authors:** Caitlin Keller, Junko Maeda, Dhileepkumar Jayaraman, Sanhita Chakraborty, Michael R. Sussman, Jeanne M. Harris, Jean-Michel Ané, Lingjun Li

**Affiliations:** ^1^Department of Chemistry, University of Wisconsin–Madison, Madison, WI, United States; ^2^Department of Agronomy, University of Wisconsin–Madison, Madison, WI, United States; ^3^Department of Bacteriology, University of Wisconsin–Madison, Madison, WI, United States; ^4^Department of Plant Biology, University of Vermont, Burlington, VT, United States; ^5^Department of Biochemistry, University of Wisconsin–Madison, Madison, WI, United States; ^6^School of Pharmacy, University of Wisconsin–Madison, Madison, WI, United States

**Keywords:** AP-MALDI, mass spectrometry imaging, *Medicago truncatula*, salt stress, metabolomics

## Abstract

Matrix-assisted laser desorption/ionization-mass spectrometry imaging (MALDI-MSI) is routinely used to determine the spatial distributions of various biomolecules in tissues. Recently, there has been an increased interest in creating higher resolution images using sources with more focused beams. One such source, an atmospheric pressure (AP) MALDI source from MassTech, has a laser capable of reaching spatial resolutions of 10 μm. Here, the AP-MALDI source coupled with a Q Exactive HF Orbitrap platform is compared to the commercial MALDI LTQ Orbitrap XL system using *Medicago truncatula* root nodules. AP-MALDI parameters, such as the S-lens value, capillary temperature, and spray voltage, were optimized on the Q Exactive-HF platform for optimal detection of plant metabolites. The performance of the two systems was evaluated for sensitivity, spatial resolution, and overall ability to detect plant metabolites. The commercial MALDI LTQ Orbitrap XL was superior regarding the number of compounds detected, as at least two times more *m/z* were detected compared to the AP-MALDI system. However, although the AP-MALDI source requires a spatial resolution higher than 10 μm to get the best signal, the spatial resolution at 30 μm is still superior compared to the 75 μm spatial resolution achieved on the MALDI platform. The AP-MALDI system was also used to investigate the metabolites present in *M. truncatula* roots and root nodules under high salt and low salt conditions. A discriminative analysis with SCiLS software revealed *m/z* ions specific to the control and salt conditions. This analysis revealed 44 *m/z* ions present at relatively higher abundances in the control samples, and 77 *m/z* enriched in the salt samples. Liquid chromatography-tandem MS was performed to determine the putative molecular identities of some of the mass ions enriched in each sample, including, asparagine, adenosine, and nicotianamine in the control samples, and arginine and soyasaponin I in the salt treated samples.

## Introduction

Matrix-assisted laser desorption/ionization (MALDI) is commonly used as a soft ionization technique to study a wide range of biomolecules. A powerful application of MALDI-mass spectrometry (MS) is the ability to determine the spatial distribution of molecules in a tissue slice by mass spectrometry imaging (MSI) (Caprioli et al., 1997). MALDI-MSI has been used to study a wide range of biomolecules, from small molecule metabolites (Lee et al., 2012; Gemperline et al., 2014), to neuropeptides (Chen and Li, 2010), and intact proteins (Chaurand et al., 2006).

More recently, atmospheric pressure-MALDI (AP-MALDI) was introduced, increasing the ease of sample preparation and allowing for analysis of volatile molecules, as the sample no longer needs to be placed under vacuum prior to analysis (Laiko et al., 2000a,b). Since then, AP-MALDI has been used to detect tryptic peptides (Schneider et al., 2005; Gudlavalleti et al., 2008; Nguyen and Russell, 2010), pesticides (Mahale et al., 2017), oligosaccharides (Chong et al., 2011), and proteolytic fragments (Grasso et al., 2007). Tandem MS has also been coupled with AP-MALDI ionization, which provides the ability to fragment molecules and use the fragmentation patterns to identify biomolecules (Mayrhofer et al., 2006; Ito et al., 2012). AP-MALDI is also capable of performing imaging experiments. The handling of samples at atmospheric pressure (AP) is an advantage of the technique, as shown by the imaging of lipids with a matrix that sublimes under higher vacuum (Jackson et al., 2018). As lipids ionize readily, multiple studies have reported imaging lipids with AP-MALDI (Schober et al., 2012; Chen et al., 2018; Desbenoit et al., 2018). Other applications of AP-MALDI-MSI include imaging of secondary metabolites in licorice rhizome (Li et al., 2014) and neuropeptides in crustaceans (Chen et al., 2018).

Recent developments in MALDI-MSI have been directed at lowering the minimum spatial resolution. Lowering spatial resolution allows for increased resolution of fine molecular features and for single-cell MALDI-MSI (Baker et al., 2017). Although historically MALDI-MSI imaging experiments have been carried out above the low μm spatial resolution requirement for single cell imaging, recently instrument advances have lowered the minimum raster step size to allow imaging at higher spatial resolution (Zavalin et al., 2013, 2014; Korte et al., 2015). AP-MALDI sources have also been introduced with optimized geometry to allow for high spatial resolution, including a scanning microprobe AP-MALDI (TransMIT GmbH, Griessen, Germany). The AP-SMALDI source has allowed for imaging of metabolites and lipids in a variety of samples at 5–20 μm spatial resolution (Schober et al., 2012; Bhandari et al., 2014, 2015; Li et al., 2014; Khalil et al., 2015).

The AP-MALDI (ng) UHR system (MassTech Inc., Columbia, MD, United States) is another AP-MALDI source capable of imaging at high spatial resolutions. The source is compact, allowing for easy and fast switching between ESI and AP-MALDI. High spatial resolution is achieved through an Nd:YAG 355 nm laser with a laser spot size of 10 μm and a maximum output frequency of 10 kHz. In the source, the sample plate is approximately 2 mm away from the heated MS inlet capillary (Kellersberger et al., 2004; Tan et al., 2004; Moskovets et al., 2016). The laser operates as a continuous raster along the rows of the sample. TARGET software (MassTech Inc., Columbia, MD, United States) controls the source settings. ImageQuest software (Thermo Fisher Scientific, Waltham, MA, United States) is used to correlate the XY coordinates to the MS spectra in the raw file to create a molecular map of target analytes distributed on the tissue section.

Matrix-assisted laser desorption/ionization-mass spectrometry imaging is now commonly used to investigate metabolite distribution in various plant tissues (Kaspar et al., 2011; Lee et al., 2012). In *Medicago truncatula* (Medicago), which forms a symbiotic relationship with rhizobia for biological nitrogen fixation, MALDI-MSI has been applied to root nodules to provide insight into the metabolites involved in biological nitrogen fixation (Ye et al., 2013; Gemperline et al., 2015). In addition to studying metabolites involved in biological nitrogen fixation, metabolite distribution changes in the root nodules due to stress can also be investigated with MALDI-MSI. Salt stress results in an energy cost to plants, as they reallocate more of their energy to physiological changes that allow continued function under stress (Munns and Gilliham, 2015). This cost of energy for plants under salt stress translates into economic costs to farmers due to reduced yields (Munns and Gilliham, 2015). The ability of legumes to form symbiotic root nodules is highly sensitive even to mild concentrations of salt that do not affect other aspects of plant growth (Soussi et al., 1998; Zahran, 1999). Consequently, identifying metabolite changes within symbiotic nodules under salt stress or non-stress conditions may help us to understand why this symbiosis is so strikingly affected by moderate levels of salt stress. Salinity tolerance in Medicago has been studied by adding NaCl and monitoring metabolite changes with activity assays (Lopez et al., 2008). Gas Chromatography-MS has also been used to investigate the metabolic profile of severe salt stress (Del-Saz et al., 2016).

Here, the ability of an AP-MALDI (ng) UHR source coupled to a high resolution accurate mass platform to study metabolites in Medicago root nodules will be investigated. As a stand-alone source that can attach to multiple instruments, the AP-MALDI (ng) UHR source is a promising alternative to a traditional dedicated MALDI source for labs that might not have the ability to obtain a dedicated MALDI platform. Thus, a study to compare the performance of the AP-MALDI (ng) UHR source to a traditional MALDI platform and to demonstrate the application of the source to investigate metabolite changes due to stress is a valuable evaluation of the performance of the source. Initially, optimized AP-MALDI MSI of root nodules was compared to MALDI-MSI of root nodules on a commercial MALDI LTQ Orbitrap XL system. The AP-MALDI system was then used to study the metabolic response to salt stress through imaging at high spatial resolution. This study analyzed the localization changes of metabolites in Medicago root nodules during salt stress.

## Materials and Methods

### Materials

2,5-Dihydroxybenzoic acid (DHB) was purchased through Acros Organics (Thermo Fisher Scientific), and α-Cyano-4-hydroxycinnamic acid (CHCA) through Sigma-Aldrich. Methanol, acetonitrile, chloroform, and formic acid were purchased through Fisher Chemical (Fisher Scientific). A Millipore system was used for double distilled water. Plain microscope slides were obtained from Fisher Scientific, and indium tin oxide coated glass slides from Delta Technologies.

### Plant Growth

Seeds of *M. truncatula* cv. Jemalong A17 were acid scarified, surface sterilized, and vernalized for two overnights at 4°C. Seedlings were germinated at room temperature and transferred to sterilized growth pouches which contained 10 ml of Modified Nodulation Medium (MNM) which was modified from Buffered Nodulation Medium (BNM) (Ehrhardt et al., 1992) with addition of 1 mM KCl containing 100 mM of sodium chloride. Plants grown only in MNM medium were used as controls. The pouches were placed in a transparent box in a growth chamber with 16 h light for 4 days. The roots were inoculated with 1 ml of *Sinorhizobium meliloti* (Rm1021) (OD_600_ of 0.1), grown for another 3 weeks and nodules were harvested for subsequent analysis.

### MSI Sample Preparation

Root nodules from control and high salt plants were trimmed from the plants with 2–4 mm of the surrounding root. Nodules were embedded in 100 mg/mL gelatin and frozen on dry ice. Nodules were sectioned at 16 um thickness on a Microm HM 525 cryostat (Thermo Fisher Scientific) at -20°C. Sections were thaw-mounted onto plain glass microscope slides for analysis on the MALDI LTQ Orbitrap XL or indium tin oxide coated glass slides for analysis on the AP-MALDI QE-HF system. A TM Sprayer (HTX Technologies, LLC, Carrboro, NC, United States) was used to apply DHB and CHCA matrix. DHB matrix (40 mg/mL in 50% methanol, 0.1% formic acid) was applied with a 24 pass TM Sprayer method (30 s dry time in between passes, 90° rotation between passes and the spacing offset in between every two passes, 3 mm spacing, 1250 velocity, 80°C temperature, and 0.05 mL/min flow rate). CHCA matrix (10 mg/mL in 70% acetonitrile, 0.1% formic acid) was applied with a 4 pass TM Sprayer method (30 s dry time in between passes, 90° rotation between passes and the spacing offset in between every two passes, 1.5 mm spacing, 1200 velocity, 75°C temperature, and 0.24 mL/min flow rate). Matrix covered samples were stored in a dry box at -20°C until analysis.

### Vacuum MALDI MSI

Matrix-assisted laser desorption/ionization-mass spectrometry imaging was performed on a MALDI LTQ Orbitrap XL (referred to as MALDI) mass spectrometer (Thermo Fisher Scientific, Waltham, MA, United States) equipped with a nitrogen laser in positive ion mode. LTQ Tune software (Thermo Fisher Scientific, Waltham, MA, United States), and Xcalibur (Thermo Fisher Scientific, Waltham, MA, United States) were used to select the imaging region and step size and the instrument parameters, respectively. The laser energy for DHB was set at either 15 or 20 μJ (later replicates needed higher laser energy to get the same signal level as earlier replicates) and the laser energy for CHCA was set at 10 μJ. Imaging was performed on three biological replicates with technical replicates at 75 μm raster step size. The mass range was set to 100–1000 *m/z* and the resolution to 60,000. Two microscans were averaged at each pixel.

### AP-MALDI MSI

Atmospheric pressure-MALDI experiments were performed on an AP-MALDI (ng) UHR ion source (MassTech Inc., Columbia, MD, United States) coupled to a Q Exactive-HF (Thermo Fisher Scientific, Waltham, MA, United States). Initially, the S-lens RF value, capillary temperature, and spray voltage parameters were optimized on-tissue. Imaging experiments were conducted in positive ion mode for 100–1000 *m/z* with 60,000 resolution, two microscans, 1E6 AGC target, 100 ms maximum injection time, 3.25 kV spray voltage, 350°C capillary temperature, and 70% for the S-lens RF value. For DHB covered sections 40% laser energy was used, and for CHCA sections 25% laser energy was used on the AP-MALDI control software. Experiments were conducted at 30 μm raster size. TARGET ng software (MassTech Inc., Columbia, MD, United States) was used to set the imaging area, raster size, and laser energy. Tune software (Thermo Fisher Scientific, Waltham, MA, United States) was used to acquire data.

### MSI Data Analysis

MSiReader software (Robichaud et al., 2013) was used to create peak lists and generate images from the data. Briefly, the interrogated zone was drawn around the nodule and root and compared to the reference zone of a matrix only area. Each technical replicate was analyzed individually in MSiReader, and all data was normalized to the total ion current. For each nodule, *m/z* in more than 15% of the total area of the interrogated zone (the root and root nodule) and less than 5% of the total area of the reference zone (matrix area) were pulled out for the MALDI data sets. *M/z* in 10% of the interrogated zone and less than 5% of the reference zone were pulled out for AP-MALDI data. The analysis was performed using a ± 5 ppm window. Low numbers for the interrogated zone percentages were selected to ensure that peaks localized to a small region of the sample (and not just peaks localized to the entire sample) were pulled out. A low percentage for the reference zone was used to have a strict cut-off for removing matrix peaks. Different interrogated region percentages were used for the two platforms due to the difference in signal intensity and number of peaks pulled out between the two. For the MALDI system, 15% was used over 10% as using 10% pulled out many more noise peaks compared to 15%. Also, as the AP-MALDI system detected hundreds of peaks (compared to the over 2,000 mass spectral peaks detected by the MALDI system), the extra *m/z* were easier to manually verify for the AP-MALDI platform. As the AP-MALDI system produced fewer images, a lower cutoff threshold was used to generate as many good images as possible. Peak lists for biological replicates were generated by combining the technical replicate peak lists and combining duplicates (*m/z* within 5 ppm). Peak lists from the three biological replicates were combined and duplicates combined (5 ppm error) to create peak lists for the two platforms with each matrix. All peak lists were manually validated by visual inspection of the resulting ion images. Peak lists were imported into SCiLS software (Bruker, Bremen, Germany) along with the data for statistical analysis. Centroid data was imported with linear interpolation at a mass accuracy of 0.0005 Da for the mass axis settings. Data was normalized to the total ion current after importing, and all analysis were performed with normalization to the total ion current. The discriminative analysis was performed using receiver operating characteristic (ROC) on both the nodules and root together and the nodule and root separately. For the analysis, individual spectra from three biological replicate nodules were used for each class. The two classes were control and salt. As the control and salt nodules are not necessarily the same size, a random subset of 500 spectra for each class was used for the analysis. A 5 ppm interval width and the validated peak lists were used for the analysis. Hypothesis tests were performed on all individual spectra of the same three biological replicates as the ROC test. The entire root nodule and root area for the control and salt samples were used for the test. Specifically, the *t*-test was used with a 5 ppm window. The peak lists generated in MSiReader was again used for the test. Principal component analysis (PCA) was performed using the mean spectra of each region (each region was drawn around the root and root nodule from a technical replicate) with a 5.0 ppm interval width, five components, and unit variance scaling.

### Sample Extraction

Approximately 100 control nodules and 100 salt treated nodules (with 2–4 mm of surrounding root) were trimmed from the plants and flash frozen. Nodules were ground with a mortar and pestle under liquid nitrogen. A methanol/chloroform/water (Milli-Q) extraction was performed by adding in order three parts methanol (600 μL), one part chloroform (200 μL), and four parts water (800 μL). Samples were vortexed briefly and centrifuged for 10 min at 5000 × *g* and 4°C. The upper aqueous layer was removed, four parts methanol were added, and the extraction was vortexed briefly. The extraction was centrifuged again at 1500 × *g* for 5 min and 4°C. The supernatant (organic layer) was removed. The aqueous and organic fractions were dried down in a speedvac and saved in a -80°C freezer prior to analysis.

### LC-MS/MS for Identifications

Aqueous samples were resuspended in optima grade water with 0.1% FA at 10 mg/mL. LC-MS/MS was performed with a Dionex Ultimate 3000 UHPLC system (Thermo Fisher Scientific, Waltham, MA, United States) equipped with a Kintex C18 column (2.1 mm internal diameter × 150 mm length, 1.7 μm particle size; Phenomenex, Torrance, CA, United States) with a corresponding guard column, and a Q Exactive MS (Thermo Fisher Scientific, Waltham, MA, United States). For separation, the column temperature was 35°C, and the mobile phases were optima grade water with 0.1% formic acid (A) and acetonitrile with 0.1% formic acid (B). A 35-min gradient at a flow rate of 0.3 mL/min with the following conditions was used: 0–5 min, held at 1% B; 5–10 min, linear gradient from 1–3% B; 10–18 min, linear gradient from 3–40% B; 18–22 min, linear gradient from 40–80% B; 22–27 min, column cleaning at 95% B; and 27–35 min, re-equilibration at 1% B. The injection volume was 4 μL, and the samples were kept at 10°C during analysis. The MS was operated in the positive ion mode with a scan range of *m/z* 100–1500 using a top five method for MS/MS. A target list, which included *m/z* more prevalent in either the control nodules or salt nodules, was used to acquire MS/MS on target *m/z*. If less than 5 *m/z* on the target list were found, then the most abundant *m/z* were chosen. The MS parameters were as follows: 70,000 resolution, 1E6 AGC, and 100 ms maximum injection time. The settings for HCD MS/MS were as follows: 35,000 resolution, 1E5 AGC, 100 ms max inject time, 15 s dynamic exclusion, and collision energies of 30, 35, and 40 for injections 1, 2, and 3, respectively. MetFrag (Ruttkies et al., 2016) was used to analyze the MS/MS results by searching the [M+H]^+^, [M+Na]^+^, and [M+K]^+^ adducts against the KEGG database with 5 ppm error tolerance. The *in silico* fragmentation was matched up to the top 20–40 experimental fragments of the MS/MS spectra at a 5 ppm and 0.01 Da tolerances. MS/MS spectra from the mzCloud high-resolution MS/MS database was used where possible to validate the MetFrag identification. For mzCloud analysis, LC-MS/MS results were loaded into Compound Discoverer software (Thermo Fisher Scientific). Briefly, raw files were aligned with adaptive curve setting with 5 ppm mass and 1.0 min retention time tolerances. Unknown compounds were detected with a 5 ppm mass tolerance, three signal to noise ratio, and 1,000,000 minimum peak intensity, and then grouped with 5 ppm mass and 0.1 min retention time tolerances. A search against the mzCloud database was then performed against all activation types with a 25 activation energy tolerance, and the intensity threshold set to true. Identifications were made if the top result in MetFrag explained almost all the major fragments and there were no other strong results in the lower scoring MetFrag results (score less than 0.8 for all other hits) or if the top result in MetFrag explained almost all the major fragments, and the compound discoverer MS/MS for this compound matched almost exactly. Arginine, soyasaponin I, asparagine, and adenosine standards were obtained to verify identifications. MS/MS parameters were the same as described for the extractions.

## Results

### AP-MALDI Parameter Optimization

**FIGURE 1:**
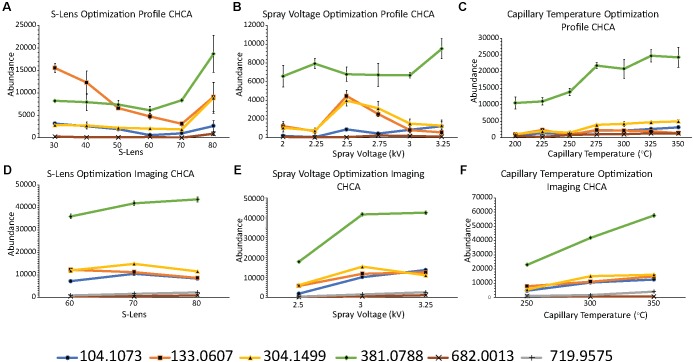
QE-HF parameter optimization graphs with CHCA as the matrix. **(A–C)** Show optimization of S-lens **(A)**, spray voltage **(B)**, and capillary temperature **(C)** by profiling on tissue. **(D–F)** Show optimization of S-lens **(D)**, spray voltage **(E)**, and capillary temperature **(F)** by imaging individual control nodules. Different *m/z* values are indicated by line color and data point shape. Error bars indicate standard error of the mean.

Initially, the AP-MALDI source and QE-HF MS parameters were optimized by metabolite profiling on tissue sections. The laser energy was optimized on matrix areas by increasing the laser energy until increasing the energy no longer increased yield of matrix ions. A wide range of S-lens, capillary temperature, and spray voltage values were tested by profiling on sections of control nodules. Ions initially enter the instrument through a heated capillary. The spray voltage is applied to the source, in this case to the plate, to assist ions into the MS. The S-lens is an ion guide behind the heated capillary consisting of a series of stacked rings that operates as a radio frequency (RF) device to capture and focus ions into a beam. Typically, larger molecules need a higher S-lens value to be efficiently transferred into the mass analyzer (Eliuk and Makarov, 2015). The temperature on the heated capillary, the voltage on the plate, and the RF value on the S-lens, all play a role in optimal detection of ions. **Figures 1A–C** shows the profiling results from adjusting the S-lens, spray voltage, and capillary temperature with CHCA as the matrix. Adjusting the S-lens and spray voltage values did not reveal any clear trend during the profiling experiments, although most *m/z* had an increase in signal at 80% for the S-lens RF value. The capillary temperature showed a stronger trend, as increasing the temperature resulted in an increased signal. **Supplementary Figures 1A–C** shows similar optimization graphs for optimizing instrument parameters with DHB as the matrix.

A smaller subset of instrument parameters was further tested by performing imaging experiments on control nodules. The base parameters were 70% S-lens, 3.0 kV spray voltage, and 300°C capillary temperature. Parameters were then individually adjusted above and below these values. **Figures 1D–F** shows the imaging results from adjusting the S-lens, spray voltage, and capillary temperature with CHCA as the matrix. **Supplementary Figures 1D–F** shows the imaging optimization results with DHB. The S-lens value still showed inconsistent results as higher *m/z* tended to increase slightly with higher S-lens while lower *m/z* decreased slightly with higher S-lens. Thus, a middle value of 70% was chosen for future experiments. In the imaging experiments, increasing the spray voltage did tend to increase the signal, especially for higher *m/z* (above 600), which were not very noticeable with the lower spray voltages. Furthermore, increasing the capillary temperature increased signal, which was consistent with the profiling results. Parameters of 3.25 kV spray voltage and 350°C capillary temperature were chosen to give the best signal, especially for higher *m/z*. The same instrument parameters were chosen for both DHB and CHCA matrices as there was not a noticeable difference between the two matrices. Spray voltages above 3.25 kV were not attempted as higher voltages could cause discharge on the AP-MALDI electronics.

α-Cyano-4-hydroxycinnamic acid matrix showed higher overall signal and better coverage of the lipid region (*m/z* above 600) compared to imaging experiments with DHB as the matrix. In **Supplementary Figure 1**, only *m/z* 104.1073 and *m/z* 133.0607 had a signal above 10,000, and this occurred at higher spray voltages and capillary temperatures. In the CHCA imaging experiments, all but the two highest *m/z* (682.0023 and 719.9575) had a signal above 10,000 at high capillary temperatures and spray voltages. In the DHB graphs in **Supplementary Figure 1**, *m/z* above 600 were not included as there was minimal signal in this region with DHB matrix.

### Comparison of AP-MALDI and Vacuum MALDI Sources

**FIGURE 2:**
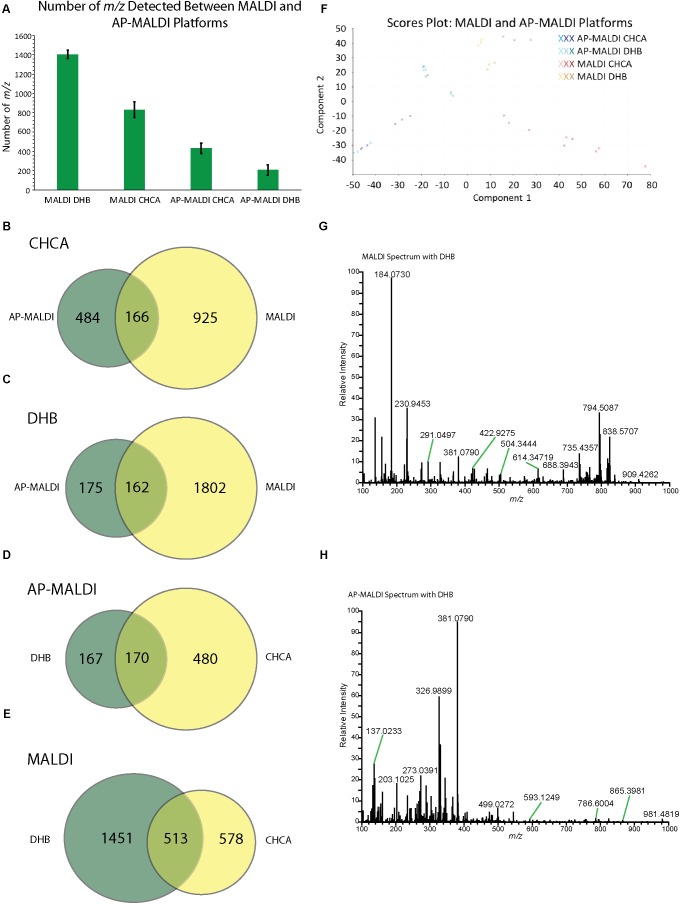
Comparison of the vacuum MALDI and AP-MALDI QE-HF systems for imaging of control nodules. In **(A)**, the number of *m/z* detected for both systems with CHCA and DHB as matrices are shown. Error bars show the standard error of the mean. **(B,C)** Show Venn diagrams for the overlap in detected *m/z* values between the two systems for CHCA **(B)** and DHB **(C)**. Venn diagrams comparing the overlap between *m/z* observed with DHB and CHCA matrices are shown for the AP-MALDI **(D)** and MALDI **(E)**. The PCA plot for all the biological and technical replicates of control nodules imaged with either the AP-MALDI or MALDI platform with either DHB or CHCA is shown in **(F)**. For each condition technical replicates are all the same color and biological replicates are differing shades of a color. Example spectra averaged over the nodule with the DHB matrix are shown for the MALDI **(G)** and AP-MALDI **(H)** platforms.

Imaging experiments using control nodules on both the AP-MALDI and MALDI platforms were conducted to compare the performance of the two instruments for MSI. **Figures 2A–H** compares the MALDI and AP-MALDI platforms. **Figure 2A** shows the number of *m/z* detected that resulted in good images (i.e., not matrix peaks and signal predominantly in plant sample) for control nodules in both the MALDI and AP-MALDI systems with both DHB and CHCA matrices. It should be noted that if a *m/z* is not detected in one platform but is detected in the other, it is likely the case that the *m/z* is present in both samples, but the signal intensity in one platform was below the threshold needed to pull out the *m/z* with MSiReader. Interestingly, DHB provided better results on the MALDI, and CHCA gave better results on the AP-MALDI system. While instrumentation differences could play a role in why different matrices were best for the two platforms, the fact that the CHCA matrix application method was a wetter method than the DHB method could also play a role. The wetter CHCA method could potentially extract metabolites form the tissue at a higher concentration. As the AP-MALDI had a lower signal than the MALDI platform, improved extraction could benefit the AP-MALDI platform more than the MALDI platform. The MALDI system detected significantly more *m/z* than the AP-MALDI system as it detected almost twice the number of *m/z* using CHCA and over six times the number of *m/z* using DHB as matrix, respectively. **Figures 2B,C** shows the overlap between the *m/z* detected in MALDI and AP-MALDI systems for DHB matrix and CHCA matrix. Both matrices had similar numbers of *m/z* shared between the two instruments. However, there were more unique *m/z* than shared *m/z*, especially for CHCA. **Figures 2D,E** compares the DHB and CHCA matrices for both the MALDI and AP-MALDI platforms. While both platforms have many *m/z* that are shared in both matrices, there is a high number of *m/z* only detected in one of the matrices, especially for DHB on the MALDI and CHCA on the AP-MALDI due to their higher number of detected *m/z* compared to the other matrix. Thus, the DHB and CHCA matrices were complementary to each other. The PCA plot in **Figure 2F** shows that the different matrix and platform conditions (MALDI DHB, MALDI CHCA, AP-MALDI DHB, AP-MALDI CHCA) all separate out into groups. The technical replicates group close together in most cases. While there is more variation in the biological replicates, the biological replicates from each matrix/platform experimental group are close enough together to separate them from the other matrix/platform experimental groups. **Figures 2G,H** shows example spectra for control nodules with DHB matrix for the MALDI (G) and AP-MALDI (H) platforms. The spectra show clear differences in *m/z* and intensity, which supports the separation of the different experimental conditions in the PCA plot. Example spectra averaged over control nodules with CHCA as the matrix for each platform are shown in **Supplementary Figure 2**. **Supplementary Tables 1, 2** list the *m/z* unique to control nodules imaged with the AP-MALDI system for DHB and CHCA, respectively. These *m/z* were compared to the matches to the mzCloud database from the LC-MS/MS data. The putative identifications from this analysis are shown in **Supplementary Table 3**. From these putative identifications, the AP-MALDI data is potentially detecting more acids as nicotinic acid/picolinic acid, pyroglutamic acid, aspartic acid, DL-α-aminosuberic acid, and pantothenic acid were all putatively identified from the *m/z* unique to AP-MALDI control nodules. Further MS/MS data collection and analysis would be necessary to verify the identity of these acids or identify additional compounds unique the AP-MALDI control nodules. The high number of unique *m/z* between the two sources is potentially due to the instrumental differences. One hypothesis for the differences is that the AP-MALDI had fewer in-source fragmentation products as other studies observed that AP-MALDI is a soft ionization method with decreased and more consistent fragmentation (Laiko et al., 2000a; Schulz et al., 2006). As the MALDI detected many more *m/z* and had a higher overall signal compared to the AP-MALDI, the *m/z* solely detected in the MALDI experiments could be due to an increased sensitivity. Also, the MALDI has a nitrogen laser, which operates at 337 nm, whereas the AP-MALDI uses an Nd/YAG laser (355 nm). The differing beam profiles of these lasers (Holle et al., 2006) could be causing some of the differences in *m/z* detected between the sources. The different efficiencies of the two instruments could also affect the detected ions. As the two platforms have two different Orbitrap instruments, the differences in ion transfer, detection, and fragmentation efficiencies can potentially result in some of the observed differences.

**FIGURE 3:**
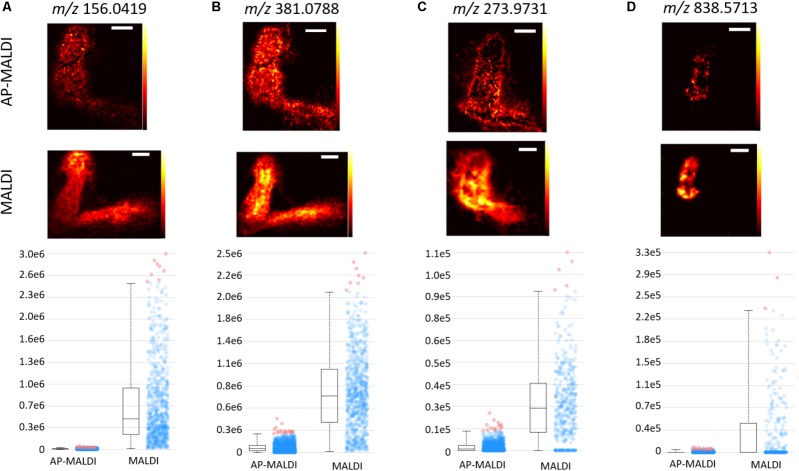
Comparison of images detected in both the AP-MALDI and MALDI platforms. Each part in **(A–D)** depicts a different *m/z* with a ±5 ppm window. For each part, the AP-MALDI image is shown on the top, the MALDI image in the middle, and the box and whisker plot on the bottom. **(A,B)** Are from the DHB matrix data and **(C,D)** are from the CHCA matrix data. The white scale bar corresponds to 1 mm.

Despite the lower number of shared *m/z* between the MALDI and AP-MALDI, the distributions of the shared *m/z* were similar. **Figure 3** compares the spatial distribution of *m/z* detected in both sources. Two representative images were selected from the shared *m/z* (see the Venn diagrams in **Figures 2B,C**) for each matrix. The *m/z* were chosen for their good normalized signal intensity in both platforms to make the images easy to compare, and an attempt was made to get an *m/z* spread evenly across the 100–1,000 range. For each *m/z*, the images acquired with the AP-MALDI have similar distributions as the images obtained on the MALDI system. However, the box and whisker plots showing the unnormalized intensity reveal a wide gap in the intensity of the signals between the AP-MALDI and MALDI. The optical images for the samples shown in **Figure 3** are shown in **Supplementary Figure 3**, and the box and whisker plots for three biological replicates of the AP-MALDI control nodules, and three biological replicates of the MALDI control nodules are shown in **Supplementary Figure 4**. After normalization to the total ion current, signals between the AP-MALDI and MALDI are much more comparable in the box and whisker plots. Although the overall signal on the AP-MALDI was lower than the MALDI, the AP-MALDI QE-HF instrument was still capable of determining the spatial distribution of small molecules in Medicago root nodules.

### Metabolites Changing Due to Salt Stress

**FIGURE 4:**
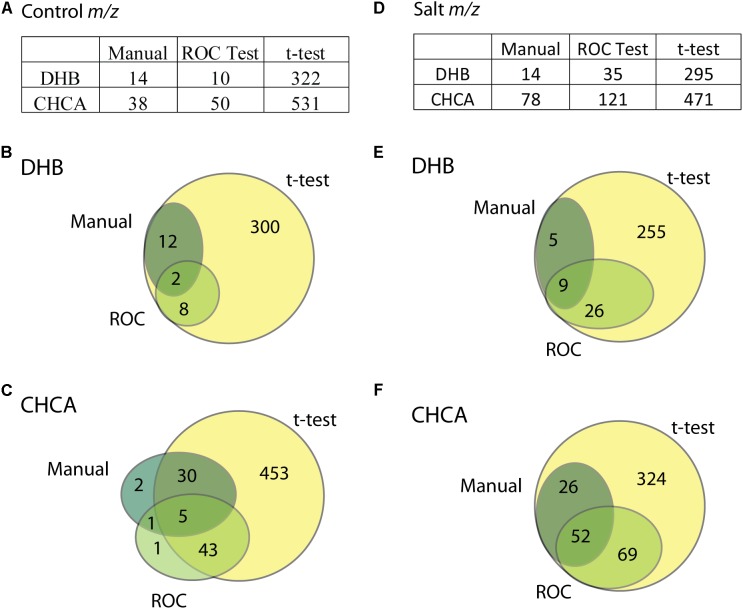
Overview of the SCiLS statistical analysis on the control versus salt nodules and roots. **(A)** Gives the number of significant *m/z* values determined for three analysis types in control nodules: manual analysis, discriminative analysis in SCiLS (ROC), and hypothesis test (*t*-test) in SCiLS software. **(B,C)** Compare the three types of analysis for DHB and CHCA matrix, respectively, using Venn diagrams. **(D–F)** Gives the same data as **(A–C)** only for significant *m/z* in the salt samples.

Overall, the quality of MS spectra obtained from salt nodules was consistent with the quality of MS spectra obtained from control nodules despite the abundance of sodium in the salt nodules. The total ion current was very similar between the control and salt nodules. For example, in one biological replicate the total ion current was 2.6E5 for control nodules versus 2.3E5 for salt nodules with CHCA and 9.6E4 for control nodules versus 4.0E4 for salt nodules with DHB. Example spectra for salt nodules with both matrices are shown in **Supplementary Figure 5** (control nodule spectra are located in **Figure 2** for DHB and **Supplementary Figure 2** for CHCA). The largest difference between the control and salt samples was the abundance of sodium adducts in the salt samples. The higher tolerance of MALDI systems to salt could potentially account for the fact that ion suppression due to the high salt concentrations in this study did not severely decrease the signal in the high salt samples. SCiLS software was used for statistical analysis of MSI data obtained from control and salt root nodules. ROC analysis was performed to generate area under the curve (AUC) values for specific *m/z*. ROC curves are generated by plotting the sensitivity (true positive rate) versus 100-specificity (false positive rate) for the ability of a single *m/z* value to discriminate between two conditions. AUC values, which range from 0 to 1, are calculated from the ROC curve for a specific *m/z*. By importing a peak list, an AUC value was generated from its respective ROC curve for each *m/z* in the list. An AUC cut-off of 0.75 was utilized as this resulted in a list of *m/z* that showed distinct differences between the control and salt nodules. As AUC values closest to 0.5 are less discriminative, an AUC value halfway in between 0.5 and 1 was chosen to give numerous *m/z* that were different between the control and salt root nodules. The ROC test was run on the entire nodule and root sample, just the nodule, and only the root to find *m/z* values that are discriminative to either the salt or control condition in specific regions of the sample (compared to *m/z* values present in the entire root and nodule sample). The discriminative analysis was compared to the *t*-test results, which were only performed on the entire root and nodule region as the *t*-test was less sensitive to the area selected. **Figure 4** compares the number of *m/z* selected from either control or salt samples using the three analysis methods: manual analysis, ROC analysis, or the *t*-test. The number of *m/z* by manual analysis was determined by looking through images for each biological replicate and selecting *m/z* that only showed signal in either the control or salt condition. The final number for the manual analysis only shows *m/z* selected in all three biological replicates.

In **Figure 4**, the results of the SCiLS analysis to find *m/z* solely in the control nodules (**Figures 4A–C**) and *m/z* strictly in the salt nodules (**Figures 4D–F**) are shown. The *t*-test found the highest number *m/z* specific to either the control or salt root nodules, with well over half of the input *m/z* having *p*-values less than 0.001. A number of these significant *m/z* did not appear to be changed in the images by naked eye, making it very hard to sort some *m/z* into either the control or salt group. The discriminative analysis test and manual analysis provided a more practical number of *m/z* to focus on. In most cases, the *m/z* selected in the manual and ROC analysis were found to be significant by the *t*-test. Differences between the manual and ROC analysis can likely be attributed to low signal in one or more biological replicates and inconsistencies in the manual sorting. Consequently, the ROC test was selected to look at the differences between the control and salt roots and root nodules. **Supplementary Table 4** lists the *m/z* and AUC values for *m/z* with AUC > 0.75 for the ROC analysis on the control roots and root nodules. **Supplementary Table 5** provides the *m/z* and AUC values for *m/z* with AUC > 0.75 in the ROC analysis on the salt roots and root nodules.

**FIGURE 5:**
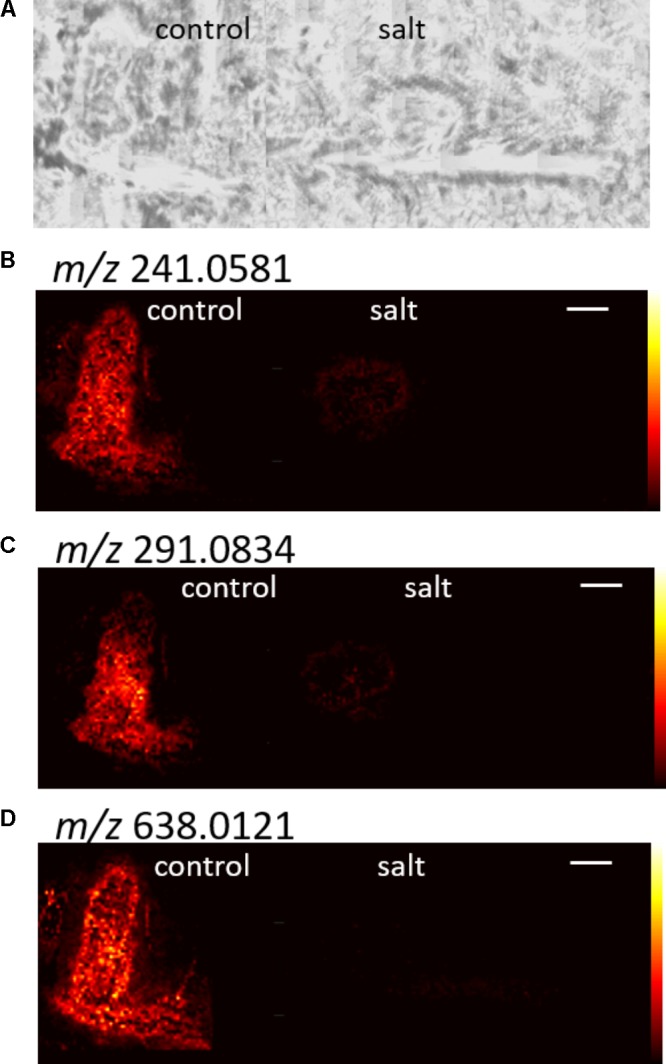
Example images for control *m/z* with AUC values above 0.75. The optical image is shown in **(A)** and **(B–D)** show three different *m/z*. CHCA was the matrix for all images shown. The white scale bar indicates 1 mm.

**FIGURE 6:**
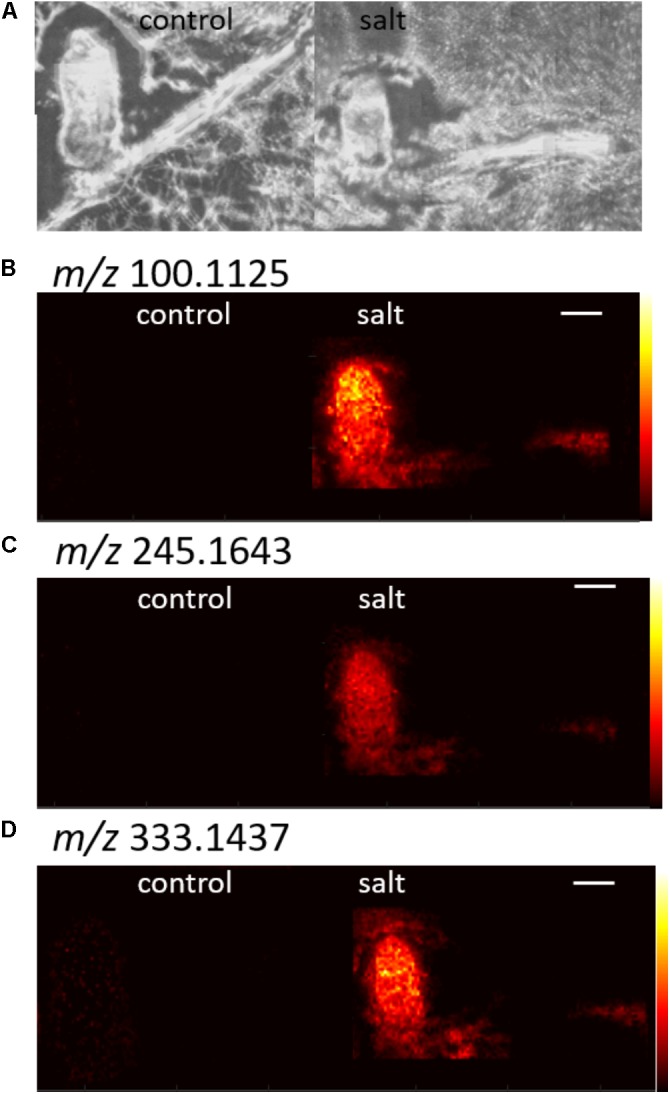
Example images for selected *m/z* ions in salt treated nodules with AUC values above 0.75. The optical image is shown in **(A)** and **(B–D)** show three different *m/z*. CHCA was the matrix for all images shown. The white scale bar indicates 1 mm.

After combining the DHB and CHCA results, removing isotope peaks, and removing images with high background signal, 44 targets from control samples, and 77 targets from salt samples were selected. Overall, a minority of images with an AUC above 0.75 were removed due to high signal in the background. **Figure 5** shows representative images from control targets, and **Figure 6** shows images for selected targets from the salt treated root nodules. Most *m/z* with an AUC higher than 0.75 show signal uniformly distributed throughout the nodule or throughout the nodule and root. Only a couple of *m/z* values, which had an AUC higher than 0.75 just in the roots, did not show any distribution in the nodule. The images show distinct differences between the control and salt nodules with AUC’s above 0.75, demonstrating the power of the ROC analysis.

**FIGURE 7:**
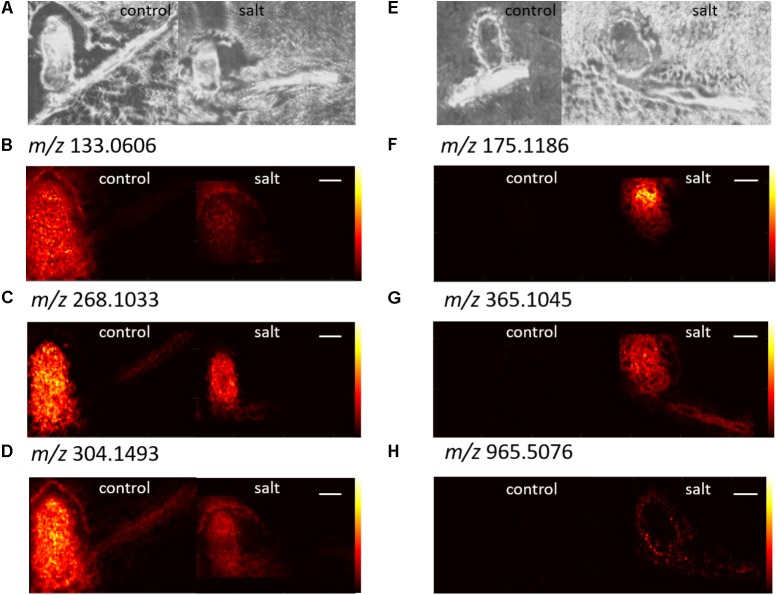
AP-MALDI MSI images for the identifications in **Table 1** **(B–D)** and **Table 2** **(F–H)**. **(A)** Shows the optical image for the control identification **(B–D)** and **(E)** shows the optical images for salt identifications **(F–H)**. Images in **(B–D,F–G)** were with CHCA as the matrix and **(H)** was with DHB as the matrix. The white scale bar corresponds to 1 mm.

Identification of metabolites from the LC-MS/MS data was performed by searching *m/z* against the KEGG database and using a combination of *in silico* fragmentation (MetFrag) and matching to the mzCloud high-resolution MS/MS database. For the MetFrag analysis, compounds that yielded theoretical fragments matching the highest number of fragments in the experimental MS/MS spectra were considered putative identifications. If more than one compound matched the major fragments, then an attempt was made to narrow down to one candidate with MS/MS spectra in the mzCloud database. **Table 1** shows the identifications from the control list of *m/z* with AUC > 0.75. Adenosine was the best option in MetFrag results, and nicotianamine was the only KEGG hit within 5 ppm for its *m/z* (the *in silico* fragmentation results did match well), but asparagine was the highest scored MetFrag result with two good options behind it. Glycylglycine was second but was ruled out with its MS/MS spectra in mzCloud. The third MetFrag result, *N*-carbamoylsarcosine, was not in mzCloud. The MS/MS spectra for asparagine in mzCloud was nearly identical to the experimental MS/MS, so it was putatively identified. Both asparagine and nicotianamine had AUC values higher than 0.75 in the nodules, while adenosine only had an AUC value higher than 0.75 in the roots, although it was also detected in the nodules. **Table 2** shows the identifications in salt roots and root nodules. Arginine was detected with an AUC higher than 0.75 in the salt nodules with MS/MS that closely matched the database spectra in mzCloud. For *m/z* 365.1045, the AUC was very high in the root nodules, roots, and roots and nodules combined, but the MS/MS was only able to distinguish the *m/z* as the sodium adduct of a disaccharide as multiple sugars ranked very high in the MetFrag analysis. Soyasaponin I was also detected as a sodium adduct and interestingly was only located to the outer portion of the root nodules and in the roots in salt nodules. **Figure 7** shows the AP-MALDI images for the *m/z* identified in **Tables 1, 2**. The experimental MS/MS spectra for the identifications are shown in **Supplementary Figure 6**. Arginine, soyasaponin I, asparagine, and adenosine experimental MS/MS spectra were compared to that of obtained standards for verification of the identification. The MS/MS spectra for the standards are in **Supplementary Figure 7**. Retention times matched closely between the experimental data and the obtained standards (values are provided in **Tables 1, 2** and **Supplementary Figure 7**).

**Table 1:** Identifications from control roots and root nodules with AUC > 0.75.

*m/z*; retention time (min)	Distribution	AUC > 0.75 location	Identification; adduct identified	Literature molecular weight	Delta ppm
133.0606; 1.05	Nodule	Nodule and root	Asparagine [M+H]^+^	132.0535	-1.48
268.1034; 3.55	Nodule and root	Root	Adenosine [M+H]^+^	267.0968	-2.53
304.1493; 1.05	Nodule and root	Nodule	Nicotianamine [M+H]^+^	303.1430	-3.31


**Table 2:** Identifications from salt treated roots and root nodules with AUC > 0.75.

*m/z*; retention time (min)	Distribution	AUC > 0.75 location	Identification; adduct identified	Literature molecular weight	Delta ppm
175.1186; 1.04	Nodule	Nodule	Arginine [M+H]^+^	174.1117	-1.81
365.1045; 1.18	Root and nodule	Root and nodule	Disaccharide [M+Na]^+^	342.1162	2.74
965.5076; 20.46	Root and outer nodule	Root	Soyasaponin I [M+Na]^+^	942.5188	-0.44


## Discussion

Here, an AP-MALDI (ng) UHR source was utilized for imaging of Medicago root nodules at 30 μm spatial resolution. The spatial resolution provided by the AP-MALDI source is much higher than the conventional MALDI, which is 75 μm spatial resolution without oversampling. The AP-MALDI source is also compatible with multiple mass spectrometers. Here, a high-resolution accurate mass QE-HF Orbitrap instrument is utilized, offering even higher mass accuracy and resolution compared to the commercial MALDI system. Furthermore, the coupling of the AP-MALDI system to a high-resolution accurate mass Orbitrap system offers distinct advantage over commercial MALDI-TOF instruments, in terms of its high mass accuracy and resolution for confident identification of small molecule metabolites.

To maximize the *m/z* detected with the AP-MALDI source, parameters were carefully optimized. The parameters selected for imaging (high capillary temperature and spray voltage) maximized the detection of most *m/z* ions. However, even with the optimized parameters, the signal in the current AP-MALDI setup was at least one order of magnitude lower than the signal with the MALDI. A previous study comparing AP and vacuum MALDI on peptides and protein digests spots revealed that although signal increased twofold in the vacuum system, the noise level increased at a similar rate, resulting in a similar signal to noise ratios between the two (Schneider et al., 2005). While a full limit of detection and signal to noise analysis was not conducted here, the MALDI detected significantly more *m/z* than the AP-MALDI, indicating a higher sensitivity for the MALDI system. The MALDI’s superior performance regarding signal intensity and detection of *m/z* provides a powerful instrument for comprehensive analysis of tissue sections. However, the lower signal did not prevent imaging of many ions with the AP-MALDI system and its higher spatial resolution provides the ability to analyze samples with fine molecular features that may be difficult to resolve with the lower spatial resolution of the MALDI instrument. In addition, the 10 kHz laser on the AP-MALDI significantly increases the speed of image acquisition compared to the 60 Hz laser on the MALDI. A 50 × 70 pixel grid on the AP-MALDI took 26.60 min to image, resulting in 2.192 pixels/s acquisition speed. However, on the MALDI, a 29 × 34 pixel grid took 65.02 min, giving an acquisition speed of 0.2527 pixels/s. Thus, the AP-MALDI is more than eight times faster than the MALDI. To acquire the 50 × 70 grid of the AP-MALDI, the MALDI would take 230.6 min compared to the 26.60 min of the AP-MALDI. Therefore, the AP-MALDI has an advantage over the MALDI system regarding the speed of acquisition. Furthermore, as at best half of the *m/z* detected with the AP-MALDI were also detected with the MALDI, the AP-MALDI-MSI results are complementary to the MALDI imaging results. The AP-MALDI source allows for the detection of additional small molecules and potentially labile small molecules that are not compatible with vacuum MALDI sources. By performing MALDI-MSI studies with both sources, one could increase the coverage of the metabolome in MALDI-MSI studies.

The AP-MALDI QE-HF system was used to study the metabolite changes due to salt stress with high spatial resolution and high mass accuracy. SCiLS software was used to perform statistical analysis on the MSI data to confidently assign *m/z* discriminative to the control and salt conditions. Although the *t*-test (*p*-value <0.001) gave the largest number of *m/z* as its output, the percentage of input *m/z* that were selected as significant was very high, and for some *m/z*, it was not apparent to the naked eye which group (either control or salt) was higher. Here, discriminative analysis using an ROC test was chosen as this test gave *m/z* with a signal that was consistently distinctive to either the salt or control group and mostly avoided *m/z* with only slight changes or changes in only one biological replicate. The discriminative analysis is also beneficial over manual analysis as it avoids potential inconsistencies in sorting. A random subset of spectra was used for the analysis as the salt nodules were typically smaller than the control nodules, meaning that using all spectra would result in a different number of spectra in each class. Although using multiple spectra per sample creates a large subset to generate ROC curves, it should be noted that individual spectra from the same sample are not independent. Furthermore, the ROC curve analysis and the *t*-test have two different meanings. The ROC curve is looking for *m/z* that discriminate between conditions (often healthy versus diseased tissue) whereas the *t*-test looking for *m/z* that have significant changes between the two conditions. While a *p*-value <0.001 and an AUC > 0.75 are not the same and provide different explanations about the data, the objective here was to compare their ability to select whichever *m/z* are changing between the conditions. As manual analysis is laborious, a statistical test to select changing *m/z* to focus identification efforts on is beneficial.

Previous studies have found changes in amino acids, organic acids, and sugars due to salt stress (Lopez et al., 2008; Del-Saz et al., 2016). Although sugars and amino acids were identified here as differing in the salt and control nodules, a potential pitfall of this study is that some of the metabolic differences observed could be due to the increased sodium levels in the salt samples. This creates a paradox observation where the same compound is higher in the control nodules for the [M+H]^+^ and [M+K]^+^ adducts, but higher in the salt treated samples for the [M+Na]^+^ adduct. The identifications of asparagine and nicotianamine in control nodule samples show this fluctuation as they had an AUC > 0.75 in the control nodules for the [M+H]^+^ adduct but the *m/z* that accurate mass matched to the sodium adduct was higher in the salt target list (MS/MS data was not able to confirm presence in salt nodules). Similarly, the disaccharide sodium adduct was identified in the salt treated samples, but based on accurate mass matching, the potassium adduct was shown upregulated in the control target list. In addition, the inability of traditional MALDI-MSI to separate isobaric compounds prevented identification of different sugars. As a result, the changes in sugar content was difficult to determine as the changes in the availability of sodium for adduction and the isobaric nature of the sugars complicated assignment significantly. However, in most cases, one can still identify metabolites changing due to salt stress (and not due to the differences in sodium adduct formation). For example, both the [M+H]^+^ and [M+Na]^+^ adducts of arginine were on the salt target list, indicating that this change is due to the effects of the stress and not due to changes in sodium availability. Although some of the compounds with AUC > 0.75 are likely due to changes in sodium levels and not due to the salt stress, there are still many targets discovered that do not show the relative intensity change between the control and salt nodules with different adducts (i.e., [M+H]^+^ adduct higher in control and [M+Na]^+^ higher in salt). These changes in the relative intensity between different adduct species can be determined by looking at the images for the control and salt nodules on the same intensity scale for each adduct species. Thus, AP-MALDI-MSI provides a viable technique to study metabolite changes in salt stress in Medicago nodules.

We observed increased accumulation of arginine in the salt-stressed nodules. Accumulation of arginine is often seen in plants subjected to various environmental stresses, and exogenous arginine helps to tolerate the harmful effects of salt stress (Rare, 1990; Kakkar et al., 2000). Arginine metabolism plays a crucial role in salt tolerance in plants as discussed below. Arginine is synthesized from the non-proteinogenic amino acid ornithine. *N*-acetylglutamate synthase (NAGS) is an enzyme that catalyzes the first reaction during ornithine biosynthesis, and overexpression of the gene encoding NAGS improves salt tolerance in tomato plants (Winter et al., 2015). Arginase catalyzes the initial reaction of arginine degradation, and a loss of activity of this enzyme is associated with increased salt tolerance, presumably via accumulation of beneficial molecules, such as, nitric oxide (NO) and polyamines (Molassiotis et al., 2010; Winter et al., 2015; Masson et al., 2017). Ornithine δ-aminotransferase, another enzyme involved in arginine catabolism shows increased activity under salt stress (Winter et al., 2015). The arginine decarboxylase (ADC) enzyme converts arginine to agmatine, which is a precursor of polyamines. Spermine is a polyamine often involved in salt tolerance, and its deficiency leads to salt hypersensitivity (Yamaguchi et al., 2006). Spermine accumulation is low in salt-treated roots in a genetic background where arginine decarboxylase activity is reduced compared to the wild-type, implicating this enzyme in salt-acclimation (Kasinathan and Wingler, 2004). In salt-tolerant rice, expression of the *ADC* gene is induced in the presence of salinity (Chattopadhyay et al., 1997). Single nucleotide polymorphisms (SNPs) associated with *ADC* showed a strong correlation with multiple environmental factors, such as, salinity, drought, and soil nitrogen, placing this enzyme as an essential regulator of plant-environment interactions (Guerrero et al., 2018). Arginine is also involved in the production of NO with the latter implicated in salt tolerance (Farooq et al., 2013; Domingos et al., 2015). Exogenous NO, in the form of its donor S-nitroso-*N*-acetylpenicillamine (SNAP), alleviates the adverse effects of salt stress, presumably by upregulating Reactive Oxygen Species (ROS)-scavenging enzymes and enhancing the accumulation of osmolytes (Ahmad et al., 2016). It is suggested that the accumulation of NO and other Reactive Nitrogen Species (RNS) cause nitrosative stress, which is essential for salt “priming” (Molassiotis et al., 2010). Altogether, these results suggest an essential position of arginine metabolism in salt stress responses.

We also found an enhanced accumulation of soyasaponin I. Saponins are amphipathic glycosides found in many plant species (Osbourn, 1996). A salt-tolerant genotype of soybean accumulates high amounts of group B saponin, alluding to its possible role in salt tolerance (Wu et al., 2008). These findings validate our technique and demonstrate that it can be used to address significant biological questions.

Here, the AP-MALDI-MSI analysis of metabolites in salt stress demonstrated the ability of the AP-MALDI (ng) UHR source to image metabolites with high resolution in both mass and space. Despite the lower number of detected compounds due to a reduced sensitivity compared to the vacuum MALDI MS platform, a respectable number of *m/z* values were found to change in root nodules between the control and salt conditions. The spatial resolution used here was not quite at the level of single-cell imaging, but with further optimization higher spatial resolutions could be achieved as the source has the potential for 5–10 μm imaging. Overall, the AP-MALDI QE-HF platform is a robust system for analyzing small molecules, and when combined with the ease of changing between AP-MALDI and ESI on a single mass spectrometer, the source makes for a useful alternative to a traditional dedicated MALDI instrument. The custom-designed source is a cost-effective substitute for a traditional MALDI platform, allowing labs to perform imaging experiments on mass spectrometers currently used with ESI. Furthermore, the complementary detection of *m/z* between the AP-MALDI and MALDI platforms allows for wider coverage of metabolites. On-going development for a new generation of a sub-AP-MALDI source from MassTech will offer improved sensitivity, and with continued ease of switching between ESI and MALDI operation, would allow for more comprehensive metabolome characterization of these important model systems.

## Author Contributions

CK performed all the experiments, sample preparation and analysis, and wrote the manuscript. SC optimized the salt stress treatment. JM grew the plants in all conditions. DJ, MS, JH, J-MA, and LL developed the research project and wrote the manuscript.

## Conflict of Interest Statement

The authors declare that the research was conducted in the absence of any commercial or financial relationships that could be construed as a potential conflict of interest.
