# Supplementary material for: Comparison of Vacuum MALDI and AP-MALDI Platforms for the Mass Spectrometry Imaging of Metabolites Involved in Salt Stress in Medicago truncatula
Source: Front Plant Sci. 2018 Aug 28;9:1238. doi: 10.3389/fpls.2018.01238 (PMC6121006; doi:10.3389/fpls.2018.01238)
Supplement: Supplementary file 1 [file Data_Sheet_1.docx]

Supplementary Material

**Comparison of Vacuum MALDI and AP-MALDI Platforms for the Mass Spectrometry Imaging of Metabolites Involved in Salt Stress in *Medicago truncatula***

Caitlin Keller^1^, Junko Maeda^2^, Dhileepkumar Jayaraman^3^, Sanhita Chakraborty^4^, Michael R Sussman^4^, Jeanne Harris^5^, Jean-Michel Ané^2,3^, Lingjun Li^1,6*^

*** Correspondence:** Dr. Lingjun Li: lingjun.li@wisc.edu

# Supplementary Figures

**Supplemental** **Figure S1**. AP-MALDI and QE-HF Parameter Optimization graphs for DHB as the matrix. **(A-C)** show optimization of S-lens **(A)**, spray voltage **(B)**, and capillary temperature **(C)** by profiling on tissue. **(D-F)** show optimization of S-lens **(D)**, spray voltage **(E)**, and capillary temperature **(F)** by imaging individual control root nodules.


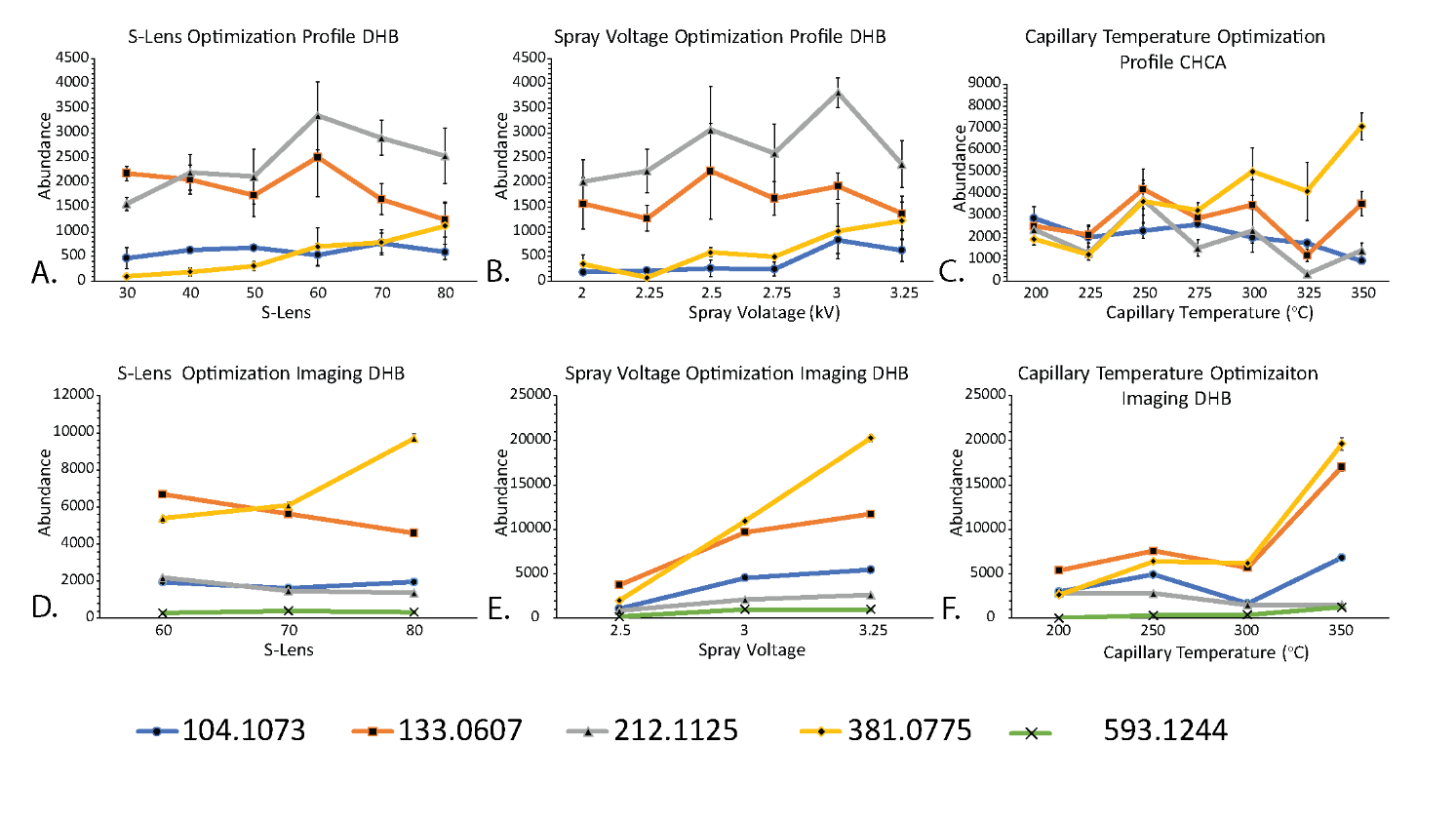


**Supplemental Figure S2.** Example spectra with CHCA as the matrix for control root nodules imaged with the MALDI platform and AP-MALDI platform. Spectra were averaged over the entire root nodule.


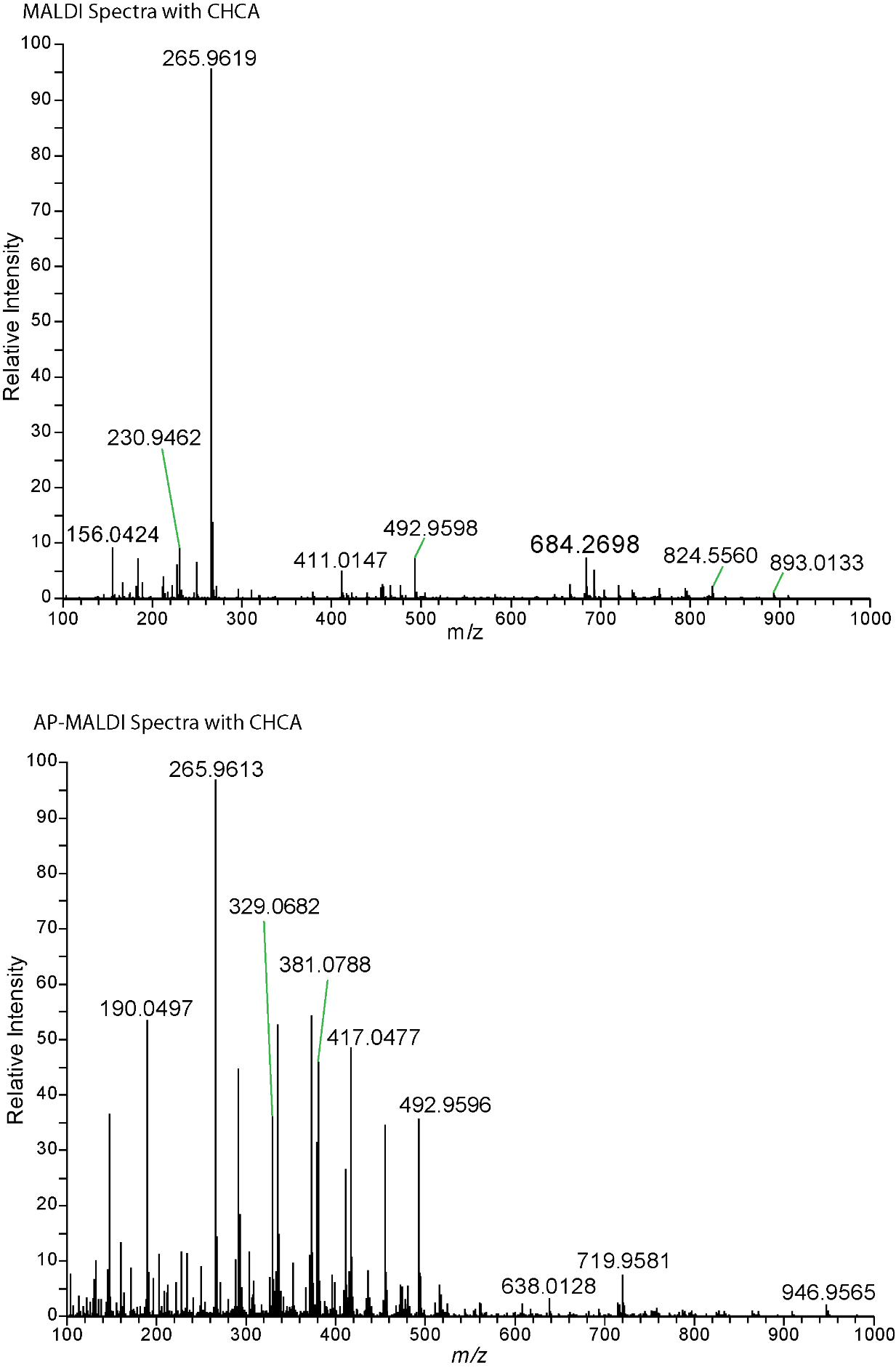


**Supplemental Figure S3.** Optical Images of Nodules from Figure 2. In **(A)**, the optical image corresponds to MALDI DHB data. In **(B)**, the optical image corresponds to AP-MALDI data, and MALDI CHCA data (serial sections of one control nodules were taken for these runs).

1.
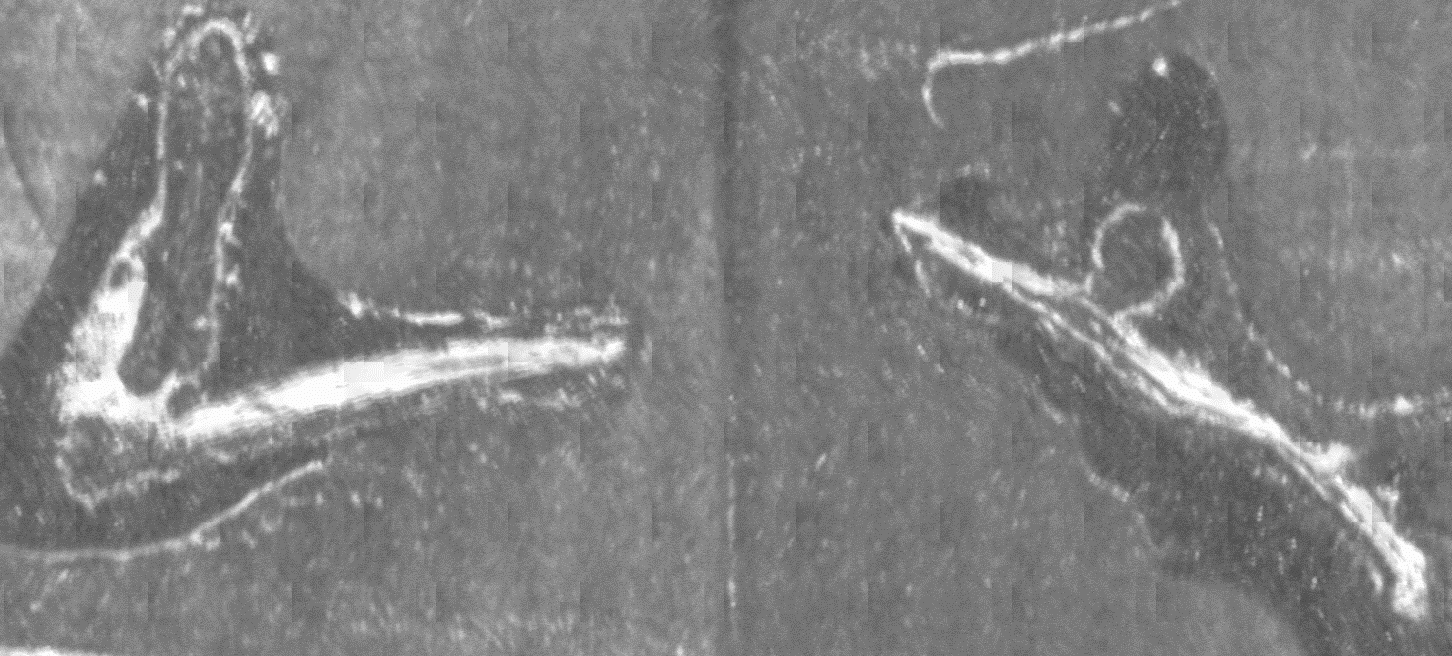
 B.
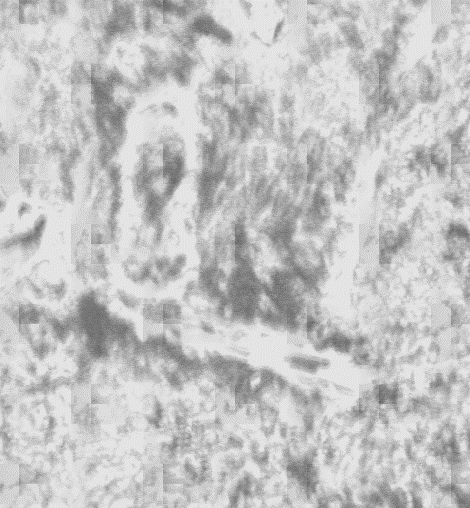


**Supplemental Figure S4.** Box and Whisker Plots comparing signal between the AP-MALDI and MALDI data. The plots show all three biological replicates for both instruments. The unnormalized data is shown in **(A,C,E,G)** and the normalized data is shown in **(B,D,F,H)**. The same *m/z* from **Figure 3** are shown. V indicates MALDI data and AP indicates AP-MALDI data.

1.
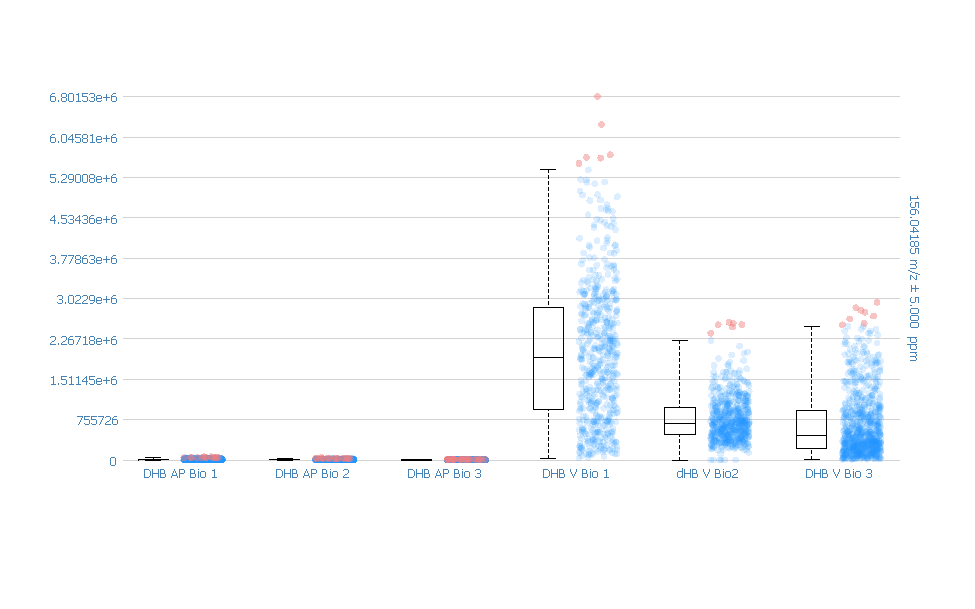

2.
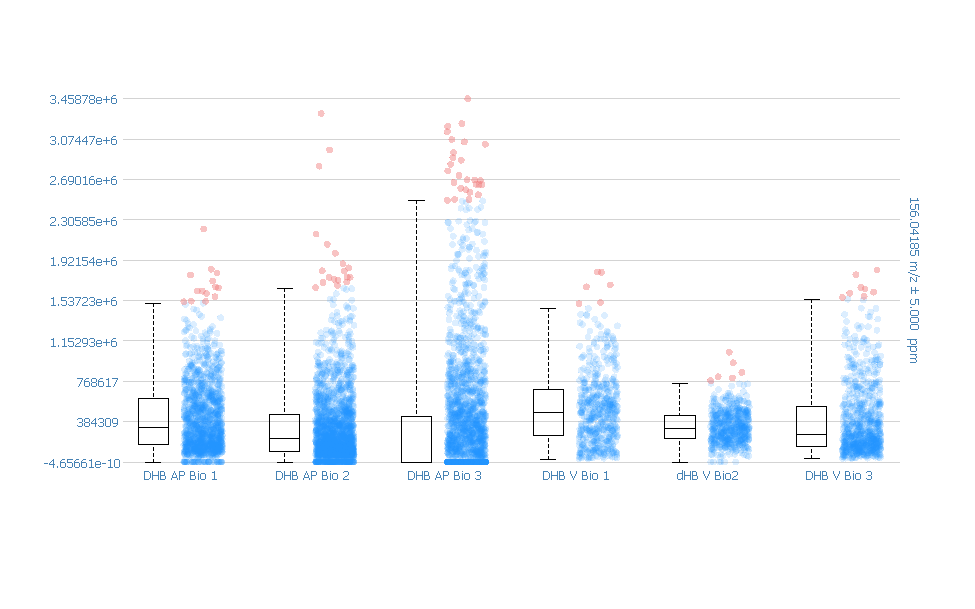

3.
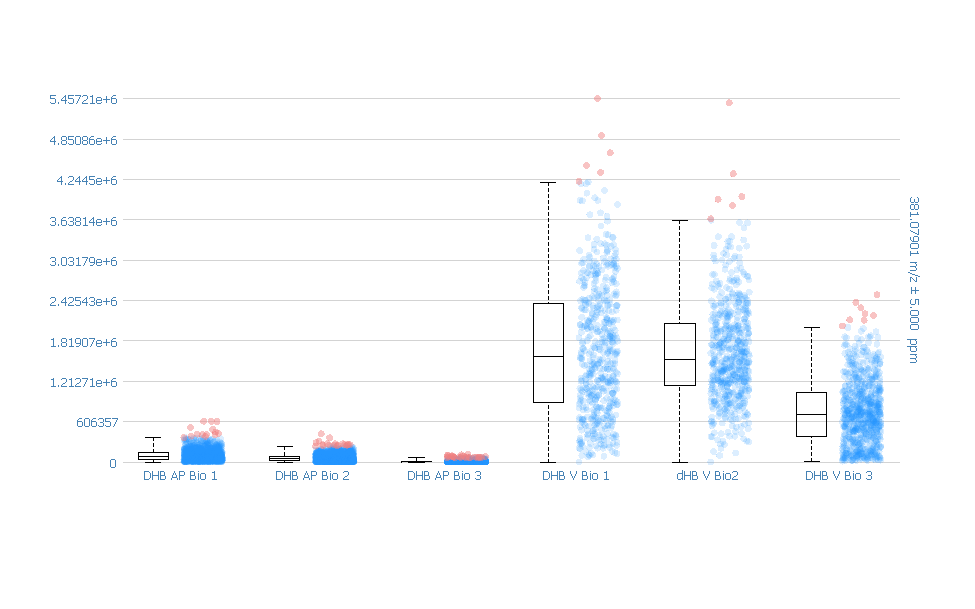

4.
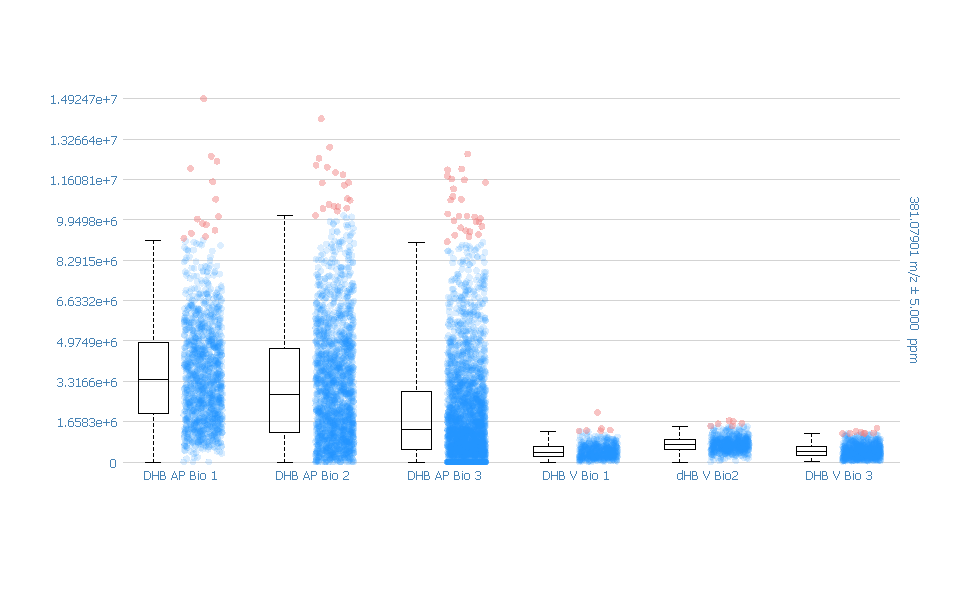

5.
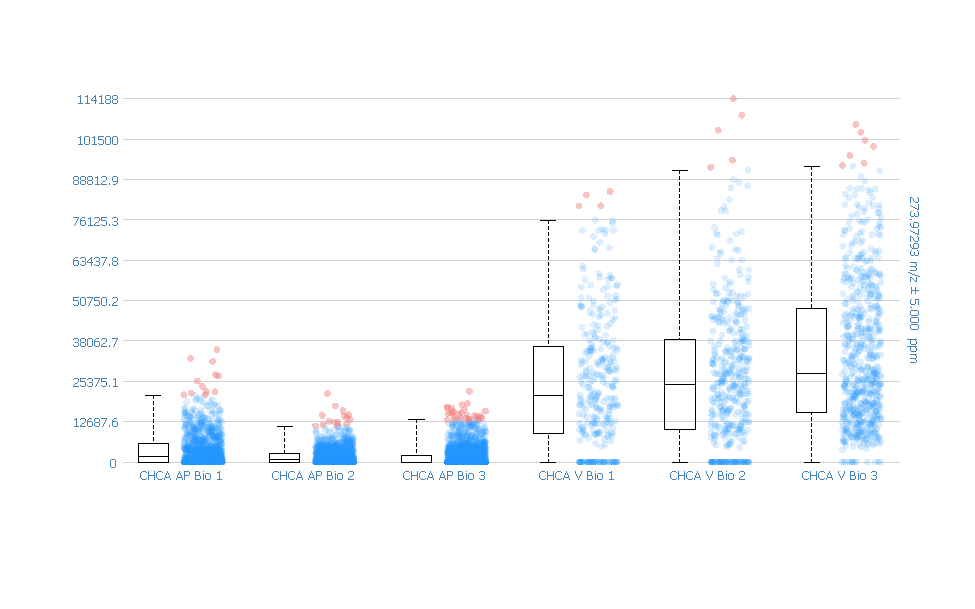

6.
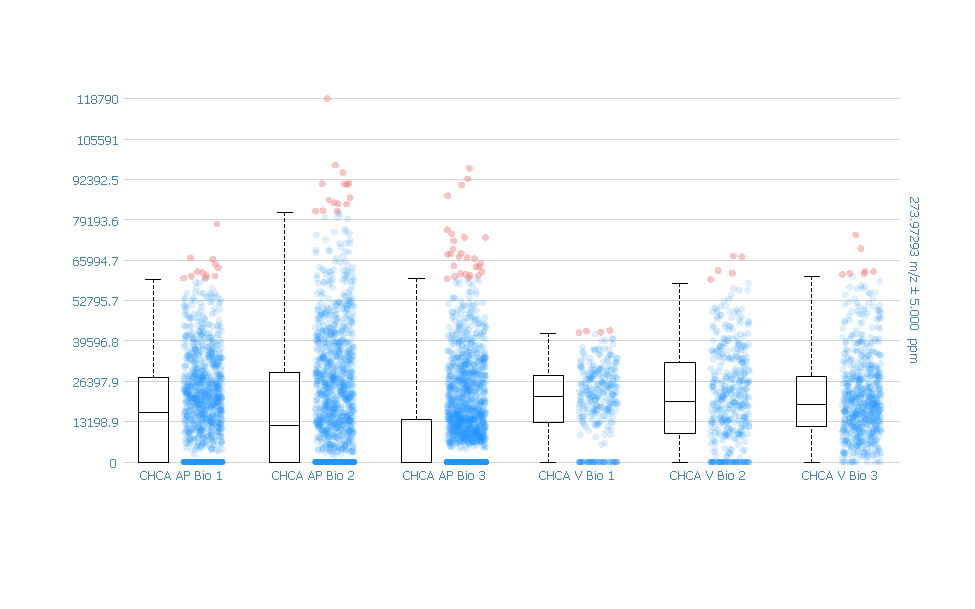

7.
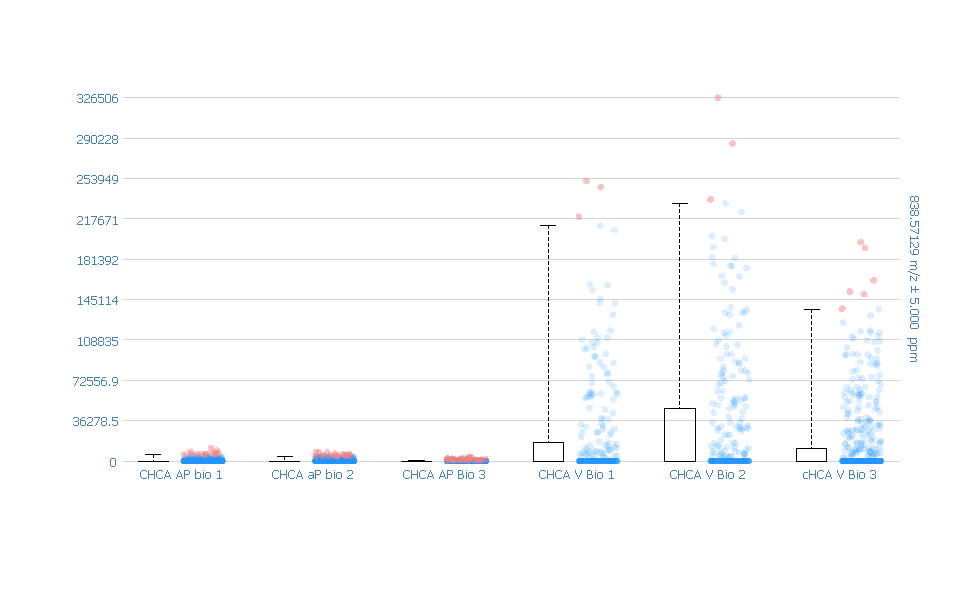

8.
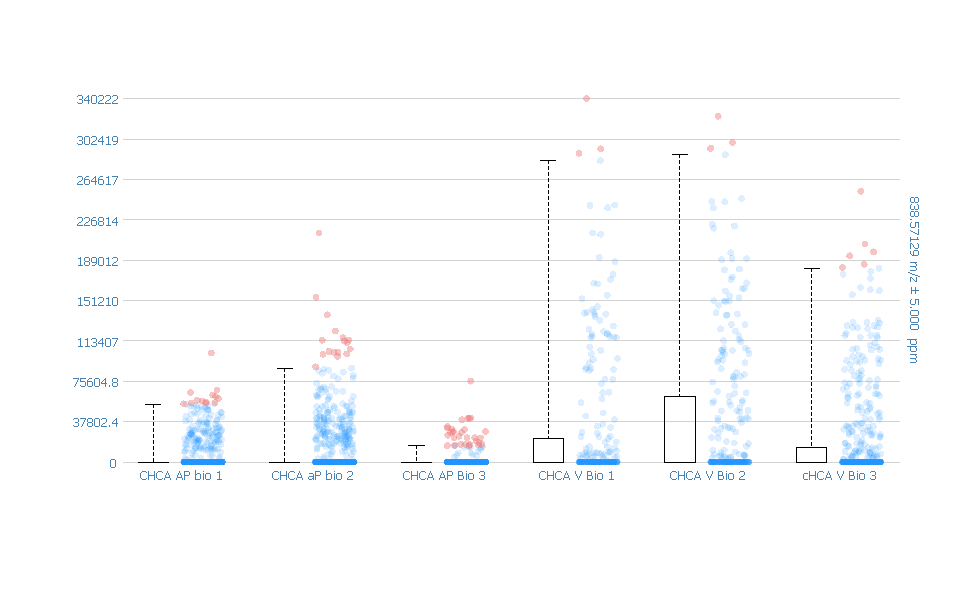


**Supplemental Figure S5.** Example spectra for salt root nodules with DHB and CHCA as the matrix. Spectra are averaged over the root nodule.

**
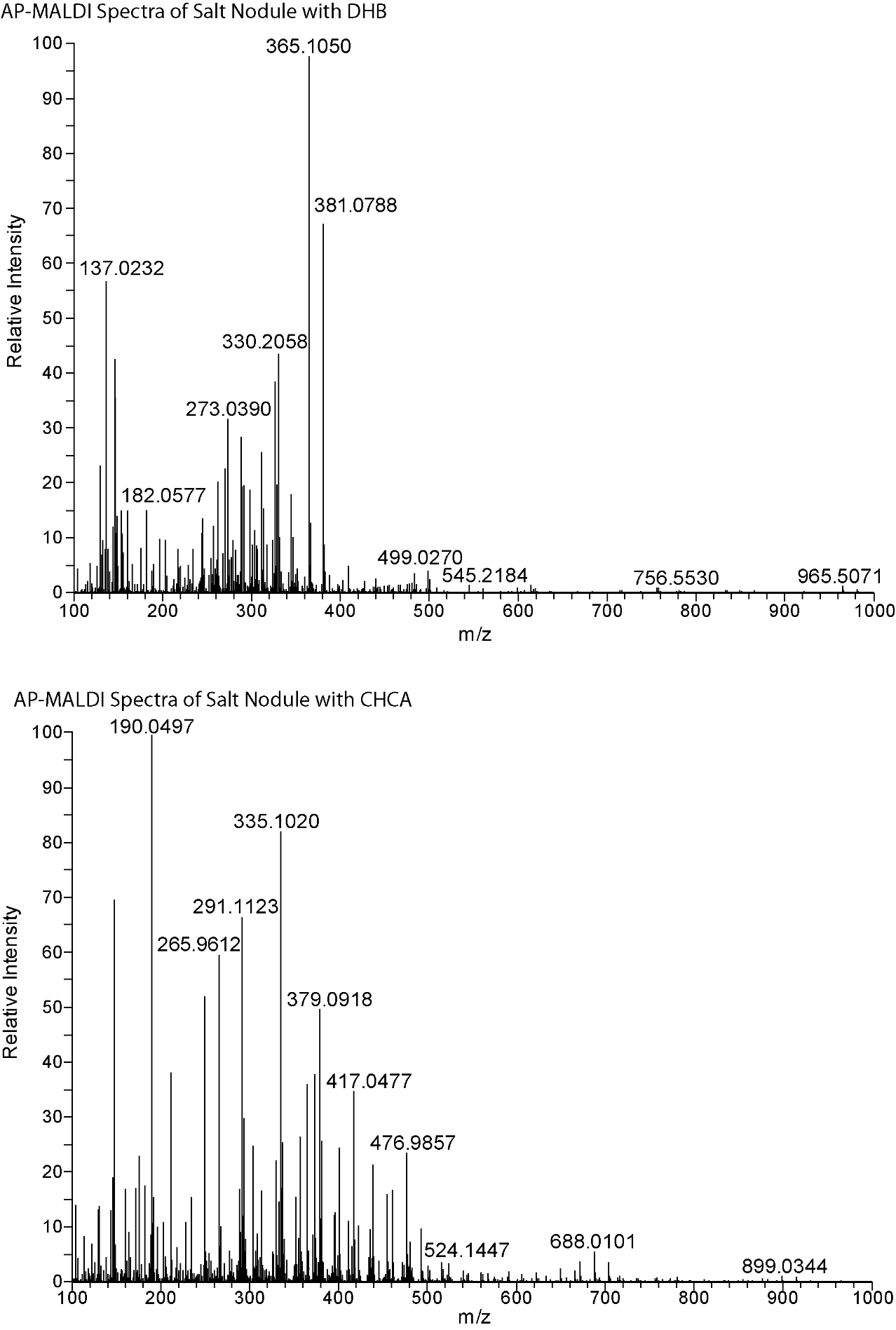
**

**Supplemental Figure S6**. MS/MS spectra used for identifications. **(A-C)** show identifications from the control root nodules in **Table 1** and **(D-F)** show identifications from the salt root nodules in **Table 2.**


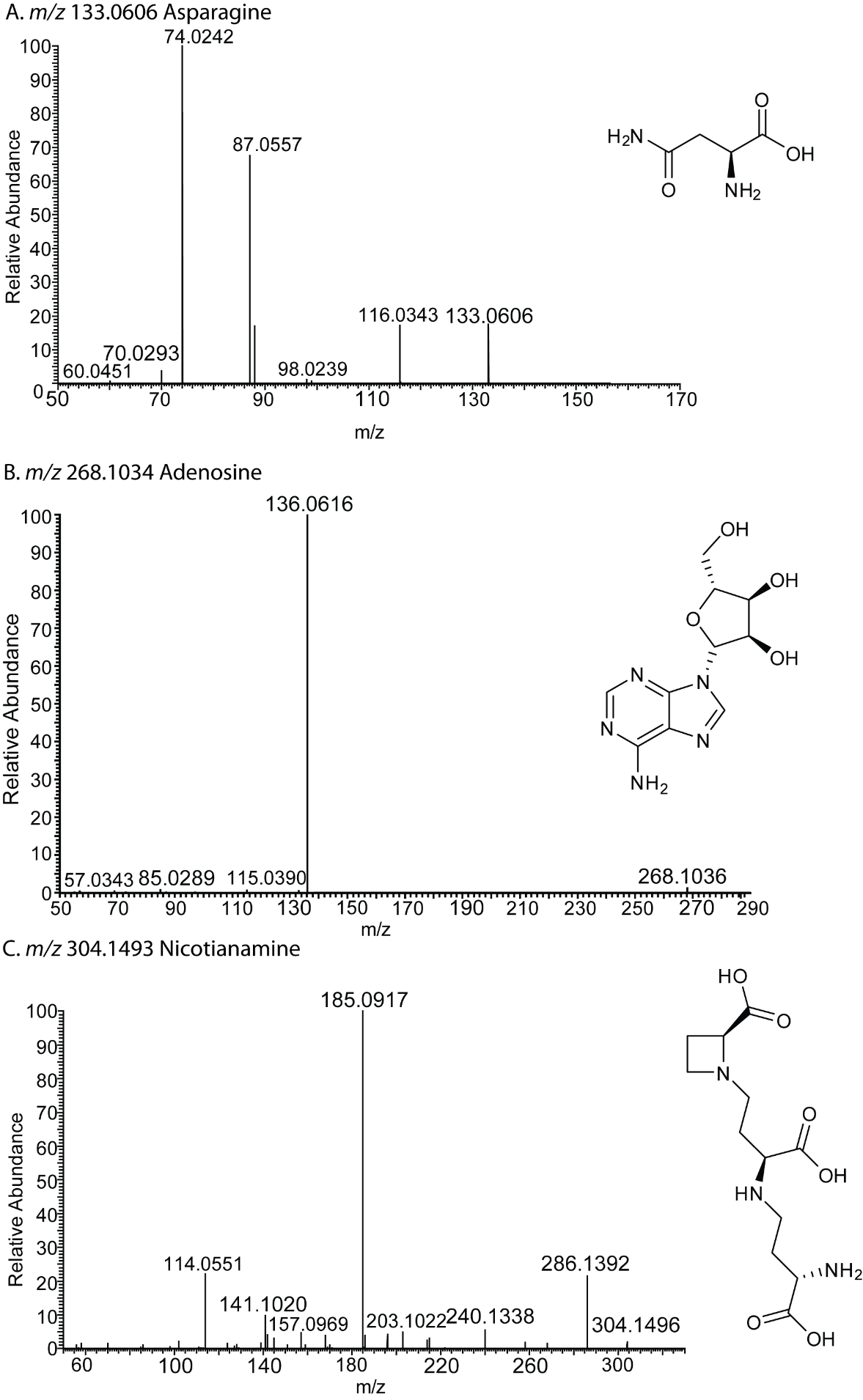


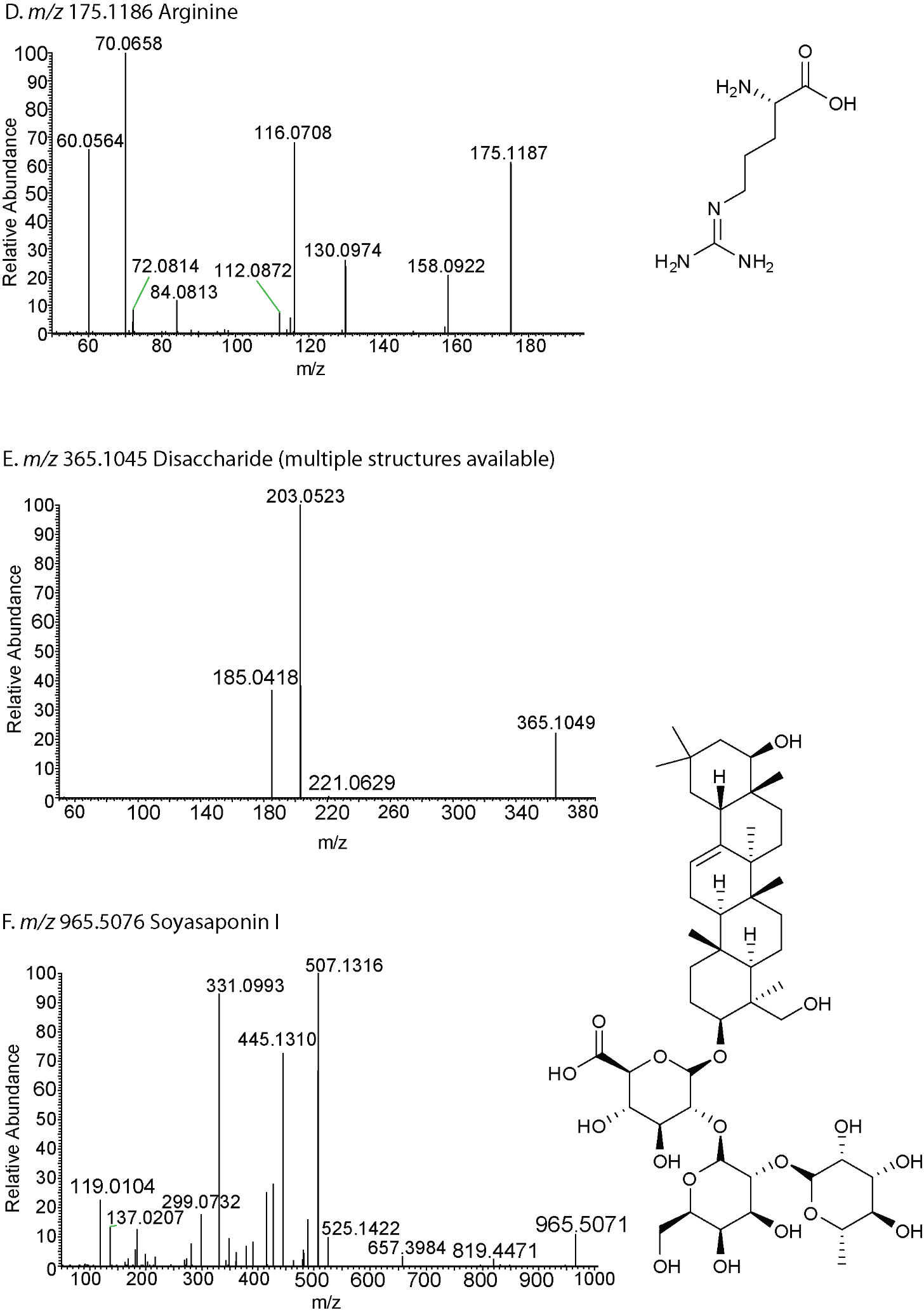


**Supplemental Figure S7.** MS/MS of standards for selected identifications. **(A)** and **(B)** are for identifications from control root nodules (asparagine and adenosine respectively) and **(C)** and **(D)** are for identifications from salt root nodules (arginine and soyasaponin I respectively).


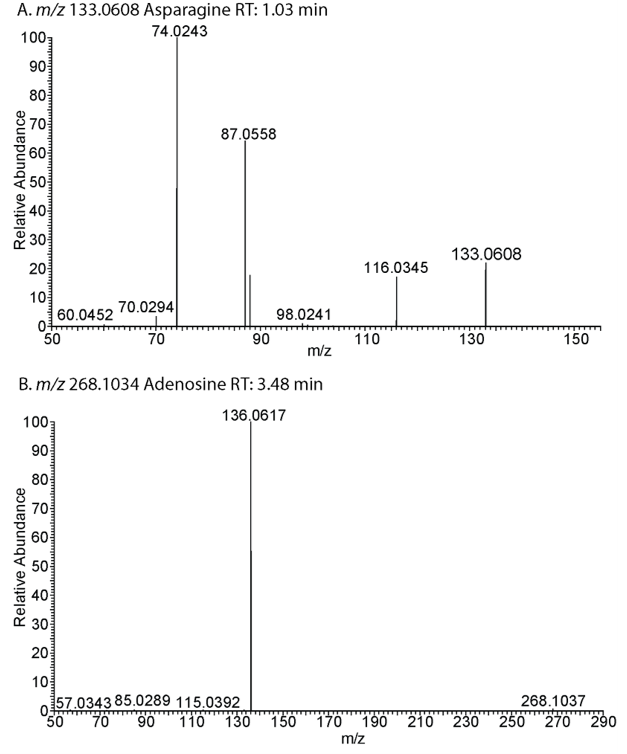


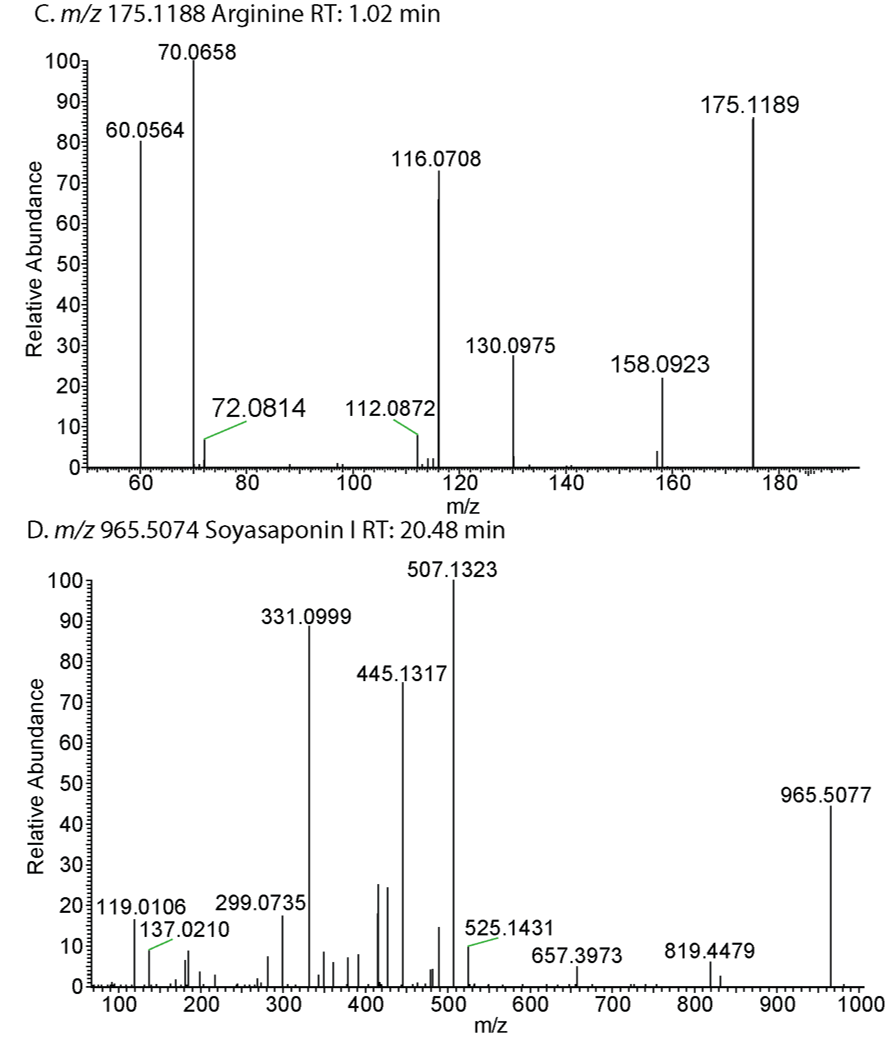


# Supplementary Tables

**Supplemental Table S1: *m/z* unique to AP-MALDI Control Nodules with DHB**

| 104.0708 | 229.9373 | 340.9816 | 398.1584 | 518.9724 |
| --- | --- | --- | --- | --- |
| 112.0874 | 230.9454 | 342.1171 | 398.9512 | 528.1338 |
| 112.1123 | 230.9903 | 342.9976 | 401.0478 | 545.2182 |
| 116.0344 | 247.1650 | 343.9917 | 404.1760 | 546.2217 |
| 120.0658 | 260.1606 | 344.1226 | 405.0925 | 547.1189 |
| 123.0552 | 268.9454 | 346.0036 | 405.1976 | 549.1352 |
| 124.0391 | 269.0762 | 349.0305 | 407.0582 | 558.9439 |
| 126.1278 | 275.1022 | 350.9663 | 410.1071 | 564.1346 |
| 127.0391 | 286.1394 | 354.9612 | 421.0875 | 577.1299 |
| 129.1387 | 288.1553 | 355.0419 | 422.0924 | 591.1094 |
| 130.0499 | 289.1397 | 357.0366 | 432.1708 | 594.1280 |
| 132.0656 | 291.1541 | 360.1496 | 436.0178 | 595.1402 |
| 134.0448 | 294.1544 | 363.9376 | 438.0316 | 601.0376 |
| 134.0640 | 294.9765 | 364.9457 | 439.0977 | 611.0624 |
| 139.0586 | 294.9998 | 365.0269 | 440.1015 | 614.0917 |
| 144.1020 | 296.1125 | 365.9530 | 440.9983 | 619.0474 |
| 146.1175 | 298.1281 | 366.1080 | 441.1135 | 621.1307 |
| 159.1127 | 299.1267 | 366.9444 | 446.1866 | 633.0265 |
| 161.0922 | 300.0022 | 366.9609 | 454.0269 | 637.0579 |
| 161.1842 | 305.1532 | 367.9519 | 455.1156 | 663.1665 |
| 162.0762 | 306.1655 | 367.9646 | 455.3515 | 707.0617 |
| 175.1077 | 307.0322 | 368.0970 | 462.9225 | 709.0772 |
| 187.1078 | 307.0785 | 368.1608 | 464.0125 | 715.5100 |
| 188.0706 | 308.0164 | 368.9594 | 465.0215 | 716.5216 |
| 188.0916 | 310.9940 | 369.1649 | 465.3343 | 725.0723 |
| 190.1072 | 311.9983 | 376.9900 | 466.0256 | 763.0278 |
| 191.1027 | 317.0052 | 378.0581 | 474.9822 | 922.6170 |
| 195.0864 | 318.1169 | 380.0090 | 475.1764 |  |
| 204.1060 | 319.0209 | 381.9484 | 476.1606 |  |
| 205.1182 | 319.1133 | 383.9597 | 476.9981 |  |
| 212.1127 | 325.1128 | 384.9547 | 490.1762 |  |
| 212.8419 | 327.9970 | 384.9717 | 490.9772 |  |
| 214.8403 | 328.1165 | 385.9751 | 491.1295 |  |
| 217.1545 | 331.0207 | 386.9874 | 492.9935 |  |
| 220.1176 | 338.9662 | 388.1808 | 498.0162 |  |
| 222.0398 | 339.9702 | 392.0970 | 509.1400 |  |
| 222.9883 | 340.1024 | 395.1073 | 517.0115 |  |

**Supplemental Table S2. *m/z* unique to AP-MALDI Control Nodules with CHCA**

| 100.1123 | 204.1057 | 284.0985 | 384.0423 | 452.9873 | 624.3857 | 787.4876 |
| --- | --- | --- | --- | --- | --- | --- |
| 104.0709 | 204.2261 | 285.0128 | 384.0804 | 453.3355 | 626.0345 | 788.0855 |
| 112.0504 | 205.0815 | 285.9432 | 385.0368 | 453.9911 | 628.8844 | 788.6091 |
| 112.0867 | 205.1181 | 286.1388 | 386.0261 | 455.3511 | 629.4537 | 789.0971 |
| 112.1123 | 205.1332 | 288.0259 | 387.0726 | 457.0188 | 635.9955 | 789.3439 |
| 114.0916 | 206.0844 | 288.1549 | 388.0401 | 458.0219 | 639.0148 | 790.1003 |
| 115.0948 | 207.1485 | 289.1392 | 390.0569 | 465.1389 | 640.0102 | 790.3482 |
| 116.0343 | 208.0965 | 289.1538 | 392.0517 | 467.1552 | 642.0284 | 795.1314 |
| 116.1070 | 210.1230 | 291.0834 | 392.0721 | 473.0886 | 644.0246 | 797.1187 |
| 120.0654 | 213.0738 | 291.0965 | 392.1592 | 474.1033 | 644.0461 | 798.1219 |
| 123.0553 | 214.1550 | 291.1698 | 394.1752 | 475.1757 | 646.3471 | 805.4128 |
| 124.0392 | 215.1023 | 293.1117 | 395.1784 | 476.1573 | 648.6280 | 813.5006 |
| 126.1278 | 216.1129 | 294.1539 | 396.1546 | 476.1852 | 649.6311 | 814.5034 |
| 127.0388 | 218.1490 | 298.1157 | 398.0137 | 482.1534 | 650.0572 | 825.0373 |
| 127.1309 | 219.0970 | 299.1265 | 398.1576 | 483.1563 | 654.1804 | 826.0421 |
| 129.0656 | 220.1175 | 301.0937 | 400.1369 | 489.0780 | 662.9688 | 827.0527 |
| 129.1386 | 221.9714 | 303.0472 | 401.0349 | 490.1361 | 664.6224 | 828.0559 |
| 130.0498 | 222.1114 | 304.1493 | 403.0650 | 490.1744 | 679.5972 | 829.0554 |
| 132.0655 | 222.9750 | 304.1650 | 404.1743 | 494.9752 | 679.9850 | 831.0730 |
| 133.0606 | 223.9694 | 305.1529 | 404.9798 | 495.9785 | 680.6010 | 833.0887 |
| 134.0444 | 223.9870 | 305.9154 | 405.0791 | 496.9737 | 682.0008 | 834.0916 |
| 134.0639 | 225.9855 | 305.9328 | 405.1149 | 496.9911 | 683.0036 | 835.0805 |
| 136.0616 | 226.0109 | 306.1548 | 407.8975 | 497.9767 | 683.9989 | 849.4024 |
| 137.0648 | 227.0628 | 306.1651 | 408.9971 | 497.9949 | 688.0153 | 851.4179 |
| 139.0579 | 230.0211 | 307.9711 | 409.0545 | 506.0121 | 693.6128 | 852.4216 |
| 141.1382 | 230.1500 | 310.8090 | 409.0784 | 509.1292 | 694.6163 | 856.0602 |
| 144.0802 | 234.0186 | 311.1127 | 409.3455 | 510.1312 | 699.1038 | 862.9948 |
| 146.0809 | 234.1233 | 313.1414 | 412.0161 | 510.9700 | 701.1188 | 865.3966 |
| 146.1174 | 235.0224 | 316.1491 | 413.0114 | 511.1447 | 702.1230 | 866.3997 |
| 146.1649 | 235.1268 | 319.0422 | 413.0283 | 512.1470 | 703.3453 | 867.0116 |
| 147.0762 | 236.0167 | 320.1805 | 414.0147 | 517.0025 | 703.9840 | 868.0132 |
| 147.1682 | 237.1016 | 321.0576 | 414.0327 | 517.1328 | 714.5057 | 869.0265 |
| 148.0598 | 238.1053 | 322.1055 | 420.1536 | 520.1471 | 715.5090 | 871.0416 |
| 149.0632 | 239.1177 | 324.0955 | 421.0778 | 526.1426 | 716.5209 | 871.4719 |
| 150.0579 | 242.0619 | 328.1166 | 421.1490 | 528.0924 | 717.5244 | 872.0449 |
| 150.0772 | 242.1495 | 329.9327 | 422.0834 | 537.1949 | 718.5353 | 873.0441 |
| 156.0763 | 243.0371 | 331.0838 | 423.1647 | 543.1319 | 719.5402 | 874.0484 |
| 157.0969 | 245.0053 | 331.1041 | 423.3609 | 545.2181 | 722.9734 | 888.1434 |
| 159.0759 | 246.0162 | 331.2122 | 424.1679 | 546.9379 | 723.9738 | 889.9673 |
| 159.1127 | 247.0571 | 332.0875 | 424.3651 | 552.9716 | 728.5221 | 893.0158 |
| 160.1805 | 247.1067 | 333.0993 | 424.9926 | 554.0502 | 730.5369 | 894.0149 |
| 161.0920 | 249.1226 | 336.2267 | 425.9968 | 555.0546 | 732.5521 | 908.9959 |
| 161.1781 | 249.1586 | 343.1082 | 429.0238 | 555.0899 | 733.5565 | 910.0017 |
| 161.1839 | 250.1909 | 343.1159 | 430.0086 | 555.2791 | 738.5058 | 910.9977 |
| 162.0759 | 252.1437 | 344.0521 | 431.0268 | 556.0672 | 740.5210 | 932.1352 |
| 172.1807 | 255.1367 | 344.1025 | 432.1694 | 556.9310 | 741.5253 | 933.1386 |
| 173.0916 | 258.0737 | 346.0464 | 433.0785 | 557.0705 | 742.5363 | 937.4175 |
| 174.0755 | 258.1095 | 346.0676 | 433.1324 | 558.0826 | 743.0919 | 947.9587 |
| 174.1967 | 258.1441 | 347.9257 | 434.1336 | 566.2024 | 743.5404 | 948.9536 |
| 175.0709 | 259.1283 | 348.0698 | 435.0577 | 568.2189 | 744.5524 | 948.9634 |
| 175.1076 | 260.1599 | 348.0830 | 435.0904 | 572.0820 | 745.1072 | 949.9581 |
| 175.1188 | 261.1225 | 348.1548 | 435.1099 | 574.1463 | 745.5557 | 949.9673 |
| 175.1438 | 263.1595 | 348.1699 | 435.9701 | 576.2209 | 746.5662 | 950.9765 |
| 177.0978 | 263.9671 | 349.0528 | 436.1486 | 576.9213 | 753.1290 | 951.9804 |
| 178.1336 | 264.2064 | 349.0939 | 436.9736 | 581.1860 | 753.4657 | 963.4711 |
| 185.0914 | 265.9610 | 349.2229 | 436.9925 | 583.1648 | 754.1328 | 979.4644 |
| 186.0759 | 266.9644 | 349.9226 | 437.1519 | 590.9266 | 755.0891 | 981.4787 |
| 186.1963 | 267.9593 | 350.1852 | 437.3407 | 592.1016 | 755.4808 | 982.4848 |
| 187.1076 | 267.9667 | 353.0842 | 437.9686 | 592.9255 | 756.4919 |  |
| 188.0546 | 267.9764 | 360.0471 | 437.9861 | 598.0398 | 759.3706 |  |
| 188.0706 | 268.1034 | 360.0607 | 438.1641 | 600.0558 | 761.5862 |  |
| 188.0909 | 268.9623 | 360.1494 | 439.1682 | 601.0597 | 772.5834 |  |
| 188.1756 | 269.1068 | 361.1518 | 439.3560 | 607.0943 | 773.5860 |  |
| 189.1343 | 269.9570 | 363.0686 | 440.1442 | 608.0769 | 775.3656 |  |
| 191.1023 | 272.0791 | 365.0083 | 440.3602 | 608.3909 | 778.4789 |  |
| 193.0970 | 278.1126 | 365.1047 | 441.8558 | 609.0801 | 781.0488 |  |
| 193.1330 | 280.1751 | 367.0235 | 443.0032 | 610.0374 | 781.1424 |  |
| 197.0664 | 280.9924 | 369.0397 | 446.1460 | 610.0771 | 783.0635 |  |
| 203.1174 | 281.9560 | 373.0300 | 446.1850 | 610.1919 | 784.0678 |  |
| 203.2226 | 282.1193 | 377.1453 | 450.1276 | 612.2075 | 785.0669 |  |
| 204.0862 | 283.9713 | 378.0934 | 450.9689 | 615.9323 | 787.0815 |  |

**Supplemental Table S3: mzCloud Putative Identifications from *m/z* unique to AP-MALDI Control Nodules**

| ***m/z*** | **Matrix** | **Compound** | **mzCloud Score** | **Theoretical *m/z*** | **Delta ppm** |
| --- | --- | --- | --- | --- | --- |
| 123.0552 | DHB | Nicotinamide | 89.3 | 123.0553 | -0.64 |
| 124.0391 | DHB | Nicotinic acid | 94.8 | 124.0393 | -1.33 |
|  |  | Picolinic acid | 85.6 |  |  |
| 127.0391 | DHB | Phloroglucinol | 87.1 | 127.0390 | 0.78 |
|  |  | 4-Hydroxy-6-methyl-2-pyrone | 85.5 |  |  |
|  |  | Maltol | 85 |  |  |
|  |  | 5-Hydroxymethyl-2-furaldehyde | 84.8 |  |  |
|  |  | Pyrogallol | 84.4 |  |  |
| 130.0499 | DHB | L-Pyroglutamic acid | 84.1 | 130.0499 | 0.15 |
|  |  | D-(+)-Pyroglutamic Acid | 83.3 |  |  |
| 132.0656 | DHB | trans-4-Hydroxy-L-proline | 87.5 | 132.0655 | 0.83 |
|  |  | cis-4-Hydroxy-D-proline | 85.9 |  |  |
|  |  | 3-Hydroxy-L-proline | 83.1 |  |  |
| 134.0448 | DHB | L-Aspartic acid | 90 | 134.0448 | 0.34 |
|  |  | D-(-)-Aspartic acid | 81.6 |  |  |
| 144.1020 | DHB | DL-Stachydrine | 91.6 | 144.1019 | 0.73 |
|  |  | 1-Aminocyclohexanecarboxylic acid | 82.5 |  |  |
| 175.1077 | DHB | N-Acetylornithine | 83.6 | 175.1077 | -0.28 |
| 190.1072 | DHB | DL-α-Aminosuberic acid | 92.4 | 190.1074 | -1.18 |
| 220.1176 | DHB | Pantothenic acid | 80.9 | 220.1179 | -1.49 |

**Supplemental Table S4**. Control *m/z* with AUC values from ROC test (AUC>0.75 is shown in bold)

| ***m/z*** | **Matrix** | **Root and Nodule AUC** | **Nod AUC** | **Root AUC** |
| --- | --- | --- | --- | --- |
| 130.0498 | CHCA | 0.6620 | **0.7589** | 0.5633 |
|  | DHB | 0.7004 | **0.8089** | 0.5480 |
| 133.0606 | CHCA | **0.7658** | **0.8240** | **0.9364** |
|  | DHB | **0.8136** | **0.8767** | **0.8125** |
| 147.0762 | CHCA | 0.7195 | **0.7878** | 0.6612 |
|  | DHB | 0.6710 | **0.7730** | 0.5501 |
| 156.0417 | CHCA | 0.6602 | **0.7834** | 0.6662 |
| 203.1021 | CHCA | 0.6999 | **0.8201** | **0.8381** |
|  | DHB | **0.7925** | **0.8774** | **0.7630** |
| 208.9722 | DHB | 0.7267 | **0.7541** | 0.6220 |
|  | CHCA | **0.7618** | **0.9130** | 0.7079 |
| 210.1230 | CHCA | 0.5777 | 0.4565 | **0.7644** |
| 212.8419 | CHCA | **0.8253** | **0.8056** | **0.9193** |
|  | DHB | 0.7372 | 0.6924 | **0.7599** |
| 217.1543 | CHCA | 0.6235 | 0.4708 | **0.8688** |
| 234.0186 | CHCA | **0.8857** | **0.8510** | **0.9278** |
|  | DHB | 0.7279 | 0.6941 | **0.8285** |
| 234.1233 | CHCA | 0.7015 | **0.7614** | 0.6093 |
| 236.0167 | CHCA | 0.6551 | 0.5439 | **0.7878** |
| 241.0581 | CHCA | 0.7087 | **0.8401** | 0.6886 |
| 247.1645 | CHCA | 0.6302 | 0.4728 | **0.8907** |
| 265.9610 | CHCA | 0.7280 | **0.8497** | 0.7238 |
| 267.9593 | CHCA | 0.7157 | **0.8170** | 0.7139 |
| 268.1034 | CHCA | 0.6700 | 0.7295 | **0.7961** |
| 271.9749 | CHCA | **0.8285** | **0.8368** | **0.8982** |
| 273.9726 | CHCA | 0.6839 | 0.6240 | **0.7800** |
| 291.0834 | CHCA | 0.7100 | **0.7826** | 0.6110 |
| 304.1493 | CHCA | 0.6541 | **0.7793** | 0.7057 |
| 342.1054 | CHCA | 0.7248 | **0.8780** | 0.6874 |
| 367.0235 | CHCA | **0.7569** | **0.7888** | 0.6173 |
| 381.0785 | CHCA | **0.7961** | **0.8339** | **0.8269** |
|  | DHB | **0.7688** | **0.7827** | 0.7149 |
| 383.0761 | CHCA | 0.6893 | 0.6784 | **0.7626** |
| 408.9971 | CHCA | **0.7999** | **0.8510** | 0.6438 |
| 413.0114 | CHCA | **0.7527** | **0.7939** | 0.6543 |
| 435.9701 | CHCA | 0.7161 | **0.8251** | 0.6023 |
| 452.9873 | CHCA | **0.7568** | **0.7930** | 0.6987 |
| 467.1552 | CHCA | 0.6906 | **0.7624** | 0.6511 |
| 475.1757 | CHCA | 0.7071 | **0.7537** | 0.6311 |
|  | DHB | 0.7342 | **0.7924** | 0.7417 |
| 492.9589 | CHCA | **0.8393** | **0.9240** | **0.8058** |
| 494.9567 | CHCA | **0.7750** | **0.8359** | **0.7868** |
| 498.9730 | CHCA | 0.6822 | 0.6566 | **0.7741** |
| 511.1447 | CHCA | 0.7052 | 0.7286 | **0.7916** |
| 608.0769 | CHCA | 0.7005 | 0.7157 | **0.7983** |
| 638.0121 | CHCA | **0.7812** | **0.8324** | 0.7416 |
| 719.9575 | CHCA | **0.8165** | **0.8750** | **0.8125** |
| 797.1187 | CHCA | 0.7286 | 0.6792 | **0.7958** |
| 871.0416 | CHCA | 0.7025 | 0.6683 | **0.8045** |
| 946.9554 | CHCA | 0.7364 | **0.7511** | 0.7287 |

**Supplemental Table S5**. Salt Sample *m/z* and AUC values from ROC test (AUC>0.75 is shown in bold)

| ***m/z*** | **Matrix** | **Root and Nodule AUC** | **Nod AUC** | **Root AUC** |
| --- | --- | --- | --- | --- |
| 100.1125 | CHCA | **0.9760** | **0.9971** | **0.9731** |
| 114.1279 | CHCA | **0.7705** | **0.8820** | 0.5946 |
| 116.0711 | DHB | 0.6468 | 0.5967 | **0.7509** |
| 120.0656 | CHCA | 0.6211 | 0.5876 | **0.7999** |
|  | DHB | 0.7137 | 0.6559 | **0.7697** |
| 129.0658 | CHCA | 0.7272 | **0.8138** | 0.6004 |
| 130.0861 | CHCA | 0.7248 | **0.8752** | 0.5149 |
| 138.1024 | CHCA | 0.6926 | 0.7222 | **0.7550** |
| 140.0681 | CHCA | **0.9255** | **0.9674** | **0.8472** |
|  | DHB | **0.7636** | 0.7303 | **0.7906** |
| 144.1016 | CHCA | **0.9615** | **0.9926** | **0.9826** |
|  | DHB | **0.8495** | **0.7944** | **0.9200** |
| 146.1173 | CHCA | **0.9317** | **0.9808** | **0.9624** |
|  | DHB | **0.9546** | **0.9807** | **0.9541** |
| 147.1128 | CHCA | 0.6716 | **0.8265** | 0.3990 |
|  | DHB | **0.8220** | **0.7762** | **0.8069** |
| 155.0424 | CHCA | **0.8203** | **0.9546** | 0.5694 |
| 158.1174 | CHCA | **0.7639** | **0.8938** | 0.5917 |
| 162.1122 | CHCA | **0.8265** | **0.8836** | 0.7294 |
| 166.0834 | CHCA | **0.9936** | **0.9892** | **0.9911** |
|  | DHB | **0.8504** | **0.8074** | **0.9236** |
| 175.1186 | CHCA | 0.7310 | **0.9085** | 0.4436 |
| 175.1439 | CHCA | 0.6729 | **0.7801** | 0.4987 |
| 177.0245 | CHCA | 0.7393 | **0.8484** | 0.5319 |
| 178.1335 | CHCA | **0.7837** | **0.8549** | **0.8081** |
| 182.0575 | CHCA | **0.9805** | **0.9928** | **0.9892** |
|  | DHB | **0.8188** | **0.7591** | **0.8969** |
| 184.0558 | CHCA | **0.8960** | **0.9014** | **0.9062** |
| 184.0731 | CHCA | 0.7368 | 0.7470 | **0.7502** |
| 188.0551 | CHCA | 0.6059 | 0.5651 | **0.8055** |
|  | DHB | 0.6374 | 0.5313 | **0.7636** |
| 189.1343 | CHCA | 0.7387 | **0.8670** | 0.5051 |
| 192.9984 | CHCA | **0.7815** | **0.9323** | 0.5397 |
| 197.1005 | CHCA | 0.6549 | **0.7785** | 0.5038 |
| 203.0523 | CHCA | **0.8095** | **0.7911** | **0.8591** |
|  | DHB | **0.7615** | 0.6737 | **0.8432** |
| 203.1499 | CHCA | 0.6804 | **0.7982** | 0.5281 |
| 203.2227 | CHCA | 0.7199 | **0.7513** | 0.6783 |
| 205.0814 | CHCA | 0.7231 | **0.7811** | **0.8106** |
|  | DHB | 0.6378 | 0.5202 | **0.7721** |
| 213.9639 | DHB | 0.7118 | 0.6421 | **0.7851** |
| 216.1128 | CHCA | 0.7138 | 0.6310 | **0.9471** |
| 217.0678 | CHCA | **0.8911** | **0.9738** | **0.7819** |
|  | DHB | **0.8319** | **0.7829** | **0.8977** |
| 217.1907 | CHCA | 0.6594 | **0.7632** | 0.5115 |
| 225.0842 | CHCA | **0.8210** | **0.9612** | 0.5783 |
| 227.0634 | CHCA | **0.8271** | **0.8022** | **0.9853** |
|  | DHB | **0.7622** | 0.6581 | **0.8910** |
| 229.0316 | CHCA | 0.6656 | **0.7677** | 0.5026 |
| 237.1018 | CHCA | 0.7110 | **0.7605** | 0.6427 |
| 240.0270 | CHCA | **0.7703** | **0.7946** | **0.7580** |
| 243.0373 | CHCA | 0.6299 | 0.6202 | **0.8381** |
|  | DHB | 0.6507 | 0.5341 | **0.7675** |
| 243.1486 | CHCA | **0.8065** | **0.8850** | 0.7113 |
| 245.1643 | CHCA | **0.9482** | **0.9888** | **0.9322** |
| 247.2010 | CHCA | **0.7651** | **0.8256** | 0.7098 |
| 249.0453 | DHB | 0.6308 | 0.5200 | **0.7528** |
|  | CHCA | 0.6303 | 0.5538 | **0.8355** |
| 252.0025 | CHCA | **0.7774** | **0.7928** | **0.8145** |
| 252.1226 | DHB | **0.7569** | 0.7182 | **0.7974** |
| 254.1382 | DHB | **0.8045** | **0.7842** | **0.8538** |
| 258.1093 | CHCA | 0.6887 | **0.7868** | 0.5977 |
| 265.0191 | CHCA | 0.7377 | 0.6720 | **0.9718** |
|  | DHB | 0.6810 | 0.5457 | **0.8356** |
| 269.0390 | CHCA | 0.7041 | **0.7926** | 0.6164 |
| 275.1097 | CHCA | 0.6852 | **0.7775** | 0.5268 |
| 280.1175 | DHB | **0.7767** | 0.7233 | **0.8407** |
| 280.9934 | CHCA | 0.5772 | 0.5141 | **0.7998** |
| 281.1490 | CHCA | **0.7611** | **0.7739** | **0.7526** |
| 283.1621 | CHCA | **0.7874** | **0.9054** | 0.6008 |
| 287.1383 | CHCA | **0.8025** | **0.8765** | 0.7014 |
| 289.1539 | CHCA | **0.9697** | **0.9959** | **0.9817** |
| 291.1695 | CHCA | **0.9047** | **0.9600** | **0.8386** |
| 298.1280 | DHB | **0.8764** | **0.8663** | **0.9165** |
| 309.1777 | CHCA | **0.8643** | **0.9366** | **0.7755** |
| 326.1314 | CHCA | **0.9047** | **0.9834** | **0.7846** |
|  | DHB | 0.7463 | 0.7332 | **0.7741** |
| 333.1437 | CHCA | **0.9297** | **0.9683** | **0.9362** |
| 335.1592 | CHCA | **0.8789** | **0.9431** | **0.7921** |
| 341.0977 | DHB | 0.6174 | 0.5056 | **0.7580** |
| 350.9867 | DHB | **0.8010** | 0.7287 | **0.8894** |
| 363.0894 | DHB | 0.6808 | 0.6157 | **0.7591** |
| 365.1045 | CHCA | **0.9677** | **0.9996** | **0.9009** |
|  | DHB | **0.8485** | **0.7890** | **0.9167** |
| 404.0224 | CHCA | 0.6581 | **0.7548** | 0.5255 |
| 408.0923 | DHB | 0.6259 | 0.5134 | **0.7692** |
| 434.2067 | CHCA | **0.7664** | **0.8212** | 0.6312 |
| 455.1151 | CHCA | **0.8399** | **0.7996** | **0.9717** |
|  | DHB | **0.7546** | 0.6837 | **0.8792** |
| 463.0089 | CHCA | **0.7660** | 0.7430 | **0.8099** |
| 576.1295 | CHCA | **0.7570** | **0.8387** | 0.7213 |
| 737.1545 | CHCA | 0.7176 | **0.7817** | 0.6549 |
| 781.1445 | CHCA | **0.7959** | **0.8534** | **0.7611** |
| 849.4227 | CHCA | 0.6876 | 0.6065 | **0.8483** |
|  | DHB | 0.6726 | 0.5646 | **0.8060** |
| 965.5076 | DHB | 0.6811 | 0.6102 | **0.7778** |
